# CLDN22 Serves as a Novel Prognostic Biomarker and Immunotherapy Response Predictor in Gliomas: A Comprehensive Multiomics Analysis

**DOI:** 10.1155/ijog/9367254

**Published:** 2025-12-20

**Authors:** Hui Zheng, Jingsong Cheng, Jialin Liu, Guodong Liu, Ronglun Dang, Rugang Luo, Jinhe Lou

**Affiliations:** ^1^ Department of Neurosurgery, The Second Affiliated Hospital of Chongqing Medical University, Chongqing, China, cqmu.edu.cn; ^2^ Department of Neurosurgery, Hami Central Hospital, Hami, Xinjiang, China; ^3^ Department of Neurosurgery, Renhuai People’s Hospital, Renhuai, Guizhou, China; ^4^ Department of Geriatrics, The Thirteenth People’s Hospital of Chongqing/Chongqing Geriatrics Hospital, Chongqing, China

**Keywords:** claudin gene, gliomas, TCGA, tumor microenvironment

## Abstract

**Background:**

The claudin gene family plays crucial roles in cancer biology, yet their comprehensive molecular characteristics and clinical implications in gliomas remain unclear.

**Methods:**

Multiomics data from The Cancer Genome Atlas (TCGA) were analyzed, and differential expression analysis was performed between glioma and normal samples. Consensus clustering was applied to identify molecular subtypes. Multiple machine learning algorithms, including least absolute shrinkage and selection operator (LASSO), extreme gradient boosting (XGBoost), Boruta, prediction analysis of microarrays (PAMR), and random forest, were employed for feature selection. Immune characteristics were evaluated using Estimation of STromal and Immune cells in MAlignant Tumors using Expression data (ESTIMATE), cell‐type enrichment analysis by gene expression signatures (xCell), and Cell‐type Identification By Estimating Relative Subsets Of RNA Transcripts (CIBERSORT) algorithms. Drug sensitivity analysis was conducted using the Genomics of Drug Sensitivity in Cancer (GDSC) database. Functional enrichment analysis was performed based on Gene Ontology (GO) terms and Kyoto Encyclopedia of Genes and Genomes (KEGG) pathways.

**Results:**

We identified distinct regulatory patterns of claudin family genes involving CNV and DNA methylation. Consensus clustering revealed two molecular subtypes with significant differences in survival (*p* < 0.001) and immune profiles. CLDN22 emerged as the most robust biomarker through machine learning integration. High CLDN22 expression correlated with poor prognosis, higher tumor grade, mesenchymal subtype, and IDH wild‐type status. CLDN22 showed superior predictive power for immunotherapy response compared to traditional biomarkers in multiple cohorts, particularly for anti‐MAGE‐A3 (AUC = 0.646), CAR‐T (AUC = 0.644), and anti‐PD‐1 (AUC = 0.646) therapies. Functional analysis revealed CLDN22′s involvement in cell adhesion, tight junction signaling, and immune cell migration. Drug sensitivity analysis identified distinct therapeutic vulnerabilities based on CLDN22 expression levels.

**Conclusion:**

Our comprehensive analysis establishes CLDN22 as a novel prognostic and predictive biomarker in gliomas with significant implications for patient stratification and therapeutic decision‐making. These findings provide new insights into glioma biology and potential therapeutic strategies, though further experimental validation is warranted.

## 1. Introduction

Glioma is the most prevalent and lethal type of primary tumor in the central nervous system (CNS) [1]. According to the World Health Organization (WHO) classification, it is classified into Grade 1 to Grade 4 inferred by its increasing malignancy. Grade 2 and 3 gliomas are classified as lower grade gliomas (LGGs), while Grade 4 is classified as higher grade gliomas (HGGs). The 2021 WHO classification of CNS tumors and the European Association of Neuro‐Oncology emphasized the vital significance of molecular features of glioma in its diagnosis, grading, and treatment approach; increasing knowledge about the genetic characteristics of different types of gliomas indicates the great era of precise medicine [2, 3]. Currently, the standard treatments of surgical resection combined with radiotherapy and pharmacotherapy have shown limited survival benefit during the past decades, and no significant breakthrough in therapeutics has been made [4]. Therefore, more effective therapeutic options are urgently needed.

Emerging evidence reveals the pivotal role of the tumor microenvironment (TME) in glioma proliferation, progression, and prognosis [5, 6]. Grossly, the immunosuppressive TME of glioma, which consists of cancer cells, stromal cells, extracellular matrix components, and various immune cells, poses significant challenges for cancer treatment by promoting tumor progression and limiting the infiltration of immune cells [7]. Efforts on transforming the TME from the “cold” one to the “hot” one would be the promising direction of future immune‐based therapeutics, such as combining several immune‐checkpoint inhibitors and other therapy modalities [8].

Claudin (CLDN) is a family of cell–cell adhesion molecules that form the tight junction and contribute to the paracellular barrier function [9, 10]. The CLDN family, which is mainly found in endothelial or epithelial cells, consists of 27 four‐transmembrane domain proteins [11]. It is revealed that the CLDN family correlates with various diseases, such as intestinal diseases and liver diseases [12–14]. In a wide variety of tumors, there are decreased or aberrant expression levels of CLDN, indicating its significant roles in carcinogenesis and metastasis. For instance, CLDN‐1 and CLDN‐7 are significantly downregulated in breast, esophageal, and prostate cancers [15]. While in colon, nasopharyngeal, ovarian, and oral squamous cell cancers, there is a corresponding overexpression of CLDN‐1. Similarly, the upregulation of CLDN‐3 and CLDN‐4 is found in ovarian, breast, gastric, pancreatic, prostate, and uterine cancers [16–18].

Our study extracted data from bulk tumors (The Cancer Genome Atlas (TCGA)) and single‐cell mRNA‐seq 23 databases. Cluster analysis was performed and CLDN22 was identified as a distinguished biomarker to explore the prognostic value and association with the glioma immune microenvironment.

## 2. Materials and Methods

### 2.1. Data Collection and Preprocessing

Transcriptome data and clinical information of patients diagnosed with GBM and LGG were downloaded from TCGA (https://www.cancer.gov/about-nci/organization/ccg/research/structural-genomics/tcga). Background correction and normalization were achieved by the robust multichip average (RMA). The fragments per kilobase million (FPKM) values were transformed into transcripts per kilobase million (TPM) values to possess a similar signal intensity with the RMA‐processed values. Then, the microarray and sequencing data were comparable as TPM values had a signal intensity similar to the RMA‐standardized values [19].

### 2.2. CLDN Family Multiomics Correlation Analysis

To comprehensively investigate the molecular characteristics of CLDN family members, we performed integrated multiomics analyses across different datasets. Pearson correlation analysis was conducted to evaluate the relationship between copy number variation (CNV) using GISTIC 2.0 analysis (https://cloud.genepattern.org) and mRNA expression levels, with results visualized using bubble plots where bubble size represented −log10(false discovery rate [FDR]) and color intensity indicated correlation strength. Somatic copy number alterations (SCNAs) were classified as amplification or deletion, and their distribution was illustrated using pie charts. DNA methylation patterns were analyzed using Spearman correlation analysis against gene expression levels, and the correlation coefficients were visualized similarly. The impact of hypermethylation on survival risk was assessed using log‐rank tests and presented as forest plots. Additionally, mutation frequencies were calculated for each CLDN gene in both LGG and GBM cohorts, and pathway activation/inhibition analysis was performed to explore the underlying molecular mechanisms.

### 2.3. Unsupervised Consensus Clustering for CLDN Family and Identified Cluster Analysis

We performed consensus clustering analysis using the ConsensusClusterPlus R package to explore molecular subtypes and their clinical significance. The optimal number of clusters (*k*) was determined by evaluating the cumulative distribution function (CDF) curves and the delta area plot, quantifying the relative change in the area under the CDF curve. A consensus matrix heatmap was generated to visualize the clustering results. Principal component analysis (PCA) was used to confirm the separation of the identified clusters in the transcriptomic space, and the results were displayed in a two‐dimensional scatter plot. Kaplan–Meier (K‐M) survival analysis was performed to compare overall survival (OS) between the clusters, and statistical significance was assessed using the log‐rank test. To further explore the genomic landscape of the identified clusters, oncoplots were generated to visualize the mutation profiles of the Top 20 most frequently mutated genes in each cluster. Mutation frequencies between clusters were compared to identify subtype‐specific genomic alterations.

### 2.4. Immunological Characteristic Analysis

Additionally, the expression algorithm (ESTIMATE) was used to estimate the stromal score, immune score, and estimate score of the infiltrating immune cells in TME. The Estimation of Stromal and Immune cells in Malignant Tumor Resource 2.0 (TIMER2.0; http://timer.cistrome.org/) web server was used to evaluate the degree of immune infiltrating cells in gliomas thoroughly. We used the xCell (cell‐type enrichment analysis by gene expression signatures) algorithm to ascertain the enrichment levels of 64 types of immune cells. The CIBERSORT (Cell‐type Identification By Estimating Relative Subsets Of RNA Transcripts) algorithm evaluated the proportions of 22 types of TME cells in tumor tissues.

### 2.5. Feature Selection and Biomarker Identification

We implemented a systematic multialgorithm integration approach to identify CLDN22 as the most predictive biomarker [20–23]. Initially, we applied five distinct machine learning algorithms for feature selection: (1) LASSO regression, utilizing 10‐fold cross‐validation to determine the optimal lambda value and selecting genes with non‐zero coefficients; (2) XGBoost, calculating feature importance scores through tree models to identify the top 4 most influential genes; (3) Boruta algorithm, determining nine significantly relevant genes by comparing with randomly permuted features within a random forest framework; (4) PAMR (Prediction Analysis for Microarrays), selecting 23 genes with optimal classification capacity through threshold filtering; and (5) random forest, identifying nine top genes based on mean decrease impurity and node splitting frequency. To ensure biomarker robustness, we exclusively considered genes consistently selected across all five algorithms. Through Venn diagram analysis, CLDN22 and SRY were identified as the only genes simultaneously meeting all algorithmic criteria. The SRY gene, located on the Y chromosome, primarily regulates male sex determination during embryonic development. It shows little or no expression in somatic tissues and lacks evidence of involvement in glioma or tumor biology. To avoid sex‐specific bias and retain only tumor‐relevant genes, SRY was excluded from downstream analyses. Subsequently, CLDN22 underwent univariate and multivariate Cox regression analyses, confirming its independent prognostic value, with its predictive accuracy validated through time‐dependent ROC curves. Furthermore, compared to traditional biomarkers, CLDN22 demonstrated superior predictive capability across multiple immunotherapy cohorts (AUC > 0.6), further substantiating its value as a key biomarker.

### 2.6. Predictive Biomarker Evaluation for Immunotherapy Response

Receiver operating characteristic (ROC) curves were constructed to assess the sensitivity and specificity of the biomarkers, and the area under the curve (AUC) was calculated for each cohort, including patients treated with anti‐PD‐1, anti‐PD‐L1, anti‐CTLA‐4, and CAR‐T therapies. For the comparative evaluation, we analyzed a range of predictive biomarkers, including tumor mutation burden (TMB), microsatellite instability (MSI) scores, gene expression signatures (e.g., CD274, IFNG, and CD8), and clonality scores (T cell and B cell clonality). AUC values were calculated for each biomarker across multiple datasets and plotted using bar graphs to visualize their predictive accuracy. Biomarker performance was compared to a random baseline (AUC = 0.5) to determine their relative effectiveness in predicting responders versus nonresponders.

### 2.7. Functional Enrichment Analysis

Gene Ontology (GO) and Kyoto Encyclopedia of Genes and Genomes (KEGG) enrichment analyses of gliomas were carried out using the R package “clusterProfiler.” GO biological process (BP), molecular function (MF), cellular component (CC) terms, and KEGG pathways with *p* < 0.05 were considered to be significantly enriched.

### 2.8. Drug Sensitivity Analysis

We performed comprehensive drug response analyses using the Genomics of Drug Sensitivity in Cancer (GDSC) database to identify potential therapeutic targets and evaluate drug sensitivity patterns. The half‐maximal inhibitory concentration (IC50) values for 198 compounds were estimated for each sample using the GDSC prediction model. Spearman correlation analysis assessed the relationship between CLDN expression levels and drug sensitivity. The correlation coefficients were visualized using bubble plots, where the size of each bubble represents the statistical significance (−log10[FDR]), and the color indicates the direction and strength of the correlation (red for positive and blue for negative correlation). We focused on FDA‐approved drugs and clinical trial compounds targeting key oncogenic pathways. Differential drug sensitivity between high and low CLDN expression groups was determined using the Wilcoxon rank‐sum test, with a FDR < 0.05 considered statistically significant.

### 2.9. Statistical Analysis

The OS of divergent groups was assessed using K‐M curves with the log‐rank test. Feature selection algorithms were implemented using the following R packages: glmnet (Version 4.1‐4) for LASSO regression, XGBoost (Version 1.5.0.2) for XGBoost analysis, Boruta (Version 7.0.0) for Boruta feature selection, PAMR (Version 1.56.1) for PAMR, and RandomForest (Version 4.7‐1) for random forest modeling. Consensus clustering was conducted using the ConsensusClusterPlus package (Version 1.58.0). Survival analyses were performed with the survival package (Version 3.3‐1) and visualized using survminer (Version 0.4.9). ROC curves and AUC calculations were generated using the pROC package (Version 1.18.0). Immune cell infiltration was estimated using immunedeconv (Version 2.0.3), which integrates CIBERSORT, ESTIMATE, and xCell algorithms. All visualizations were created using ggplot2 (Version 3.3.5), pheatmap (Version 1.0.12), and ComplexHeatmap (Version 2.10.0). Functional enrichment analyses were performed using clusterProfiler (Version 4.2.2). All statistical analyses maintained a significance threshold of *p* < 0.05. R 4.1.2 was used to conduct all statistical analyses. Statistics were considered significant when *p* value < 0.05.

## 3. Results

### 3.1. Identification and Functional Annotation Analysis of CLDN Family Genes in GBM and LGG

We revealed that CNV and methylation were key regulatory mechanisms of CLDN family gene expression in gliomas. Significant positive correlations were observed between CNV and mRNA expressions for several CLDN genes, such as CLDN12 and CLDN15, in both the GBM and LGG cohorts (Figure 1a). Heterogeneous CNV amplifications were prevalent in CLDN11, CLDN15, and CLDN14, whereas deletions were more frequently observed in CLDN3 and CLDN4 (Figure 1b,c). Additionally, methylation levels of genes such as CLDN3 and CLDN11 were negatively correlated with gene expression, particularly in LGG (Figure 1d). Survival analysis indicated that hypermethylation of CLDN22 and CLDN19 was associated with poorer prognosis in LGG (Figure 1e), while CLDN14 and CLDN11 exhibited higher mutation frequencies in both GBM and LGG (Figure 1f). Pathway enrichment analysis demonstrated that specific CLDN genes, such as CLDN14 and CLDN11, regulated key pathways, including apoptosis, cell cycle, and DNA damage repair, suggesting their potential roles in glioma progression (Figure 1g).

Figure 1(a) Pearson correlation between CNV and mRNA expressions for CLDN genes in LGG and GBM. (b) Heterogeneity of CNV types (amplification and deletion) across CLDN genes in LGG and GBM. (c) Pie charts illustrating the distribution of SCNA types for each CLDN gene in GBM and LGG. (d) Spearman correlation between methylation levels and gene expression for CLDN genes. (e) Impact of hypermethylation on overall survival risk in LGG, with circle size indicating the log‐rank *p* value significance. (f) Mutation frequency of each CLDN gene in the GBM and LGG cohorts, represented as percentages. (g) Heatmap showing CLDN gene influence on various biological pathways, with activation and inhibition effects.

**Figure:**
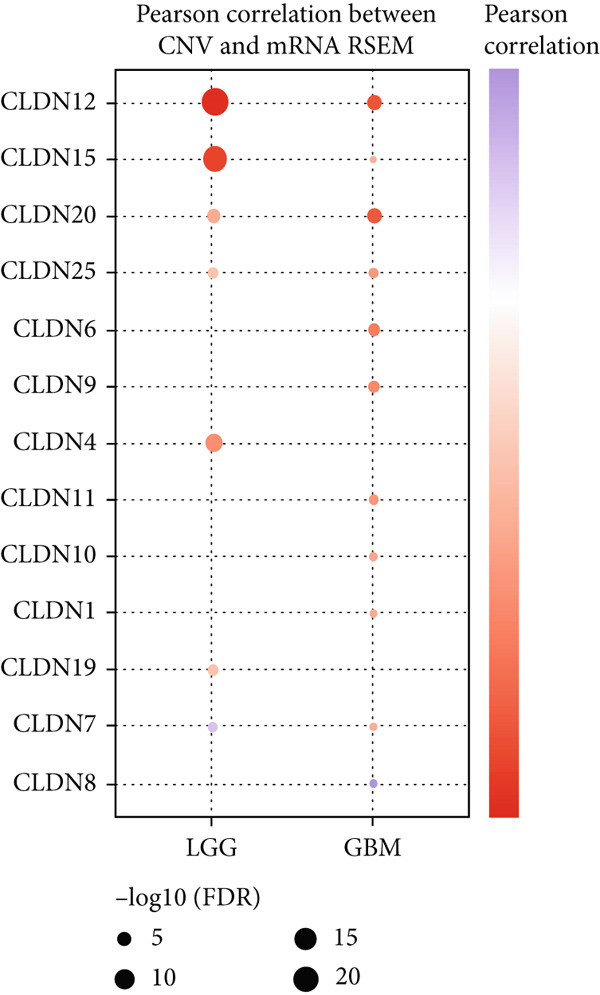
(a)

**Figure:**
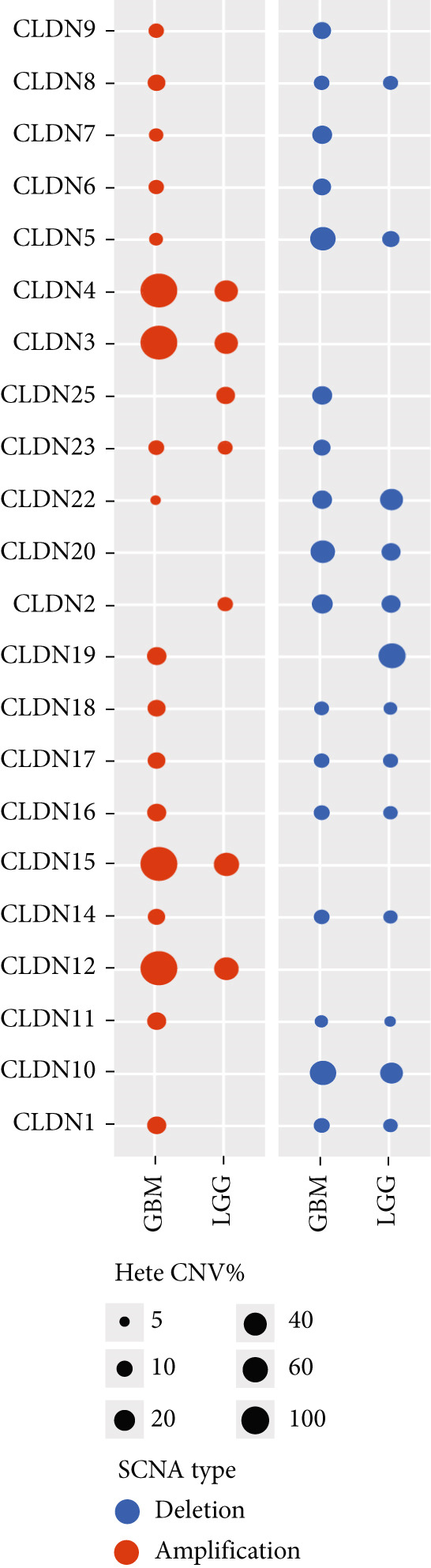
(b)

**Figure:**
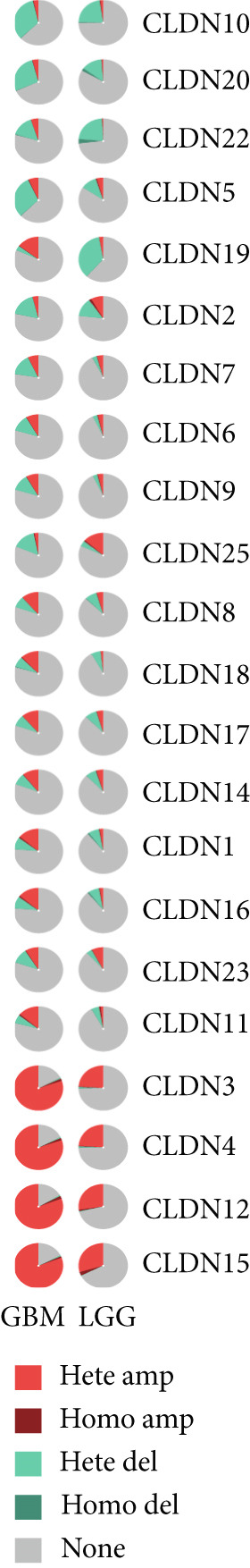
(c)

**Figure:**
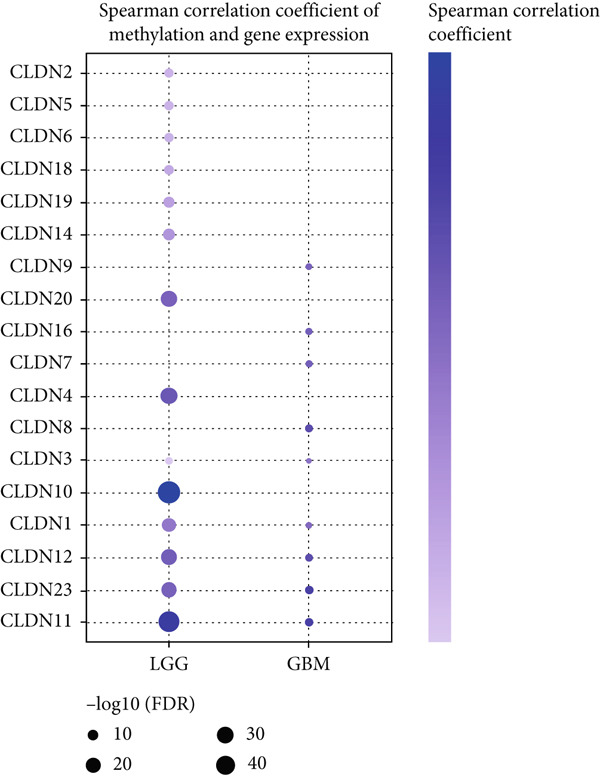
(d)

**Figure:**
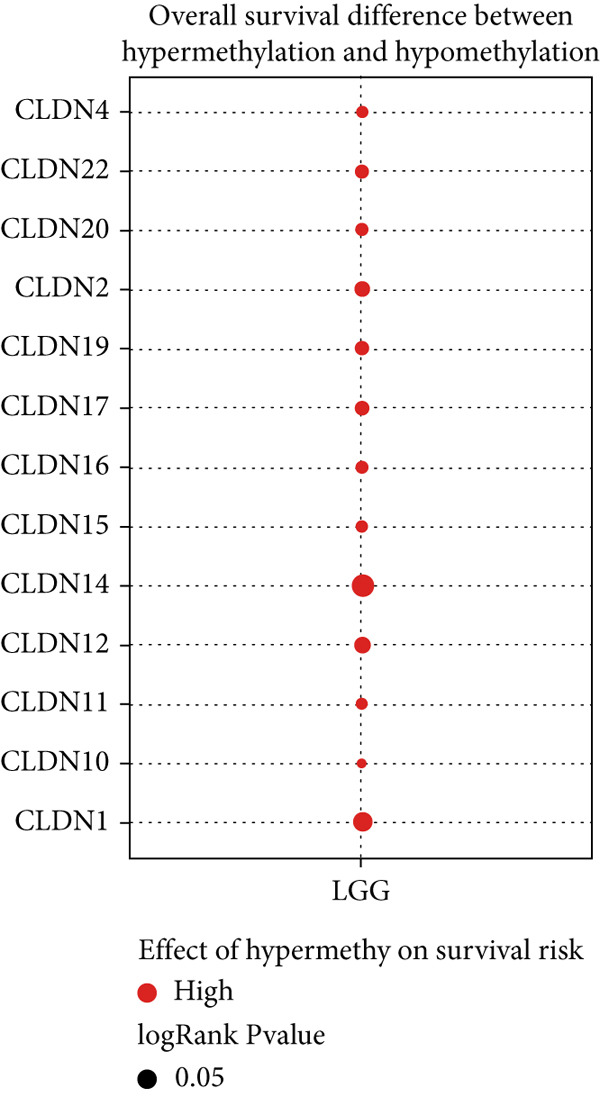
(e)

**Figure:**
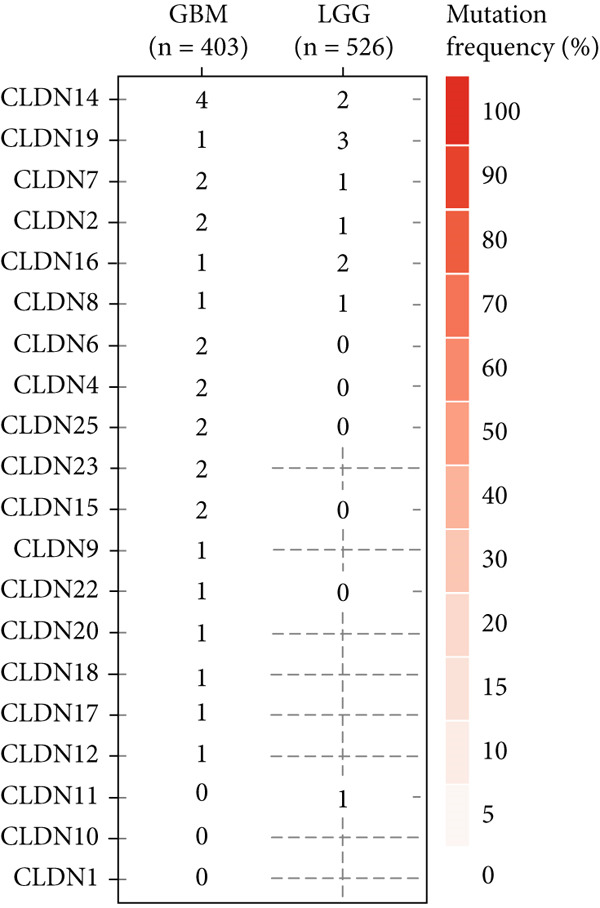
(f)

**Figure:**
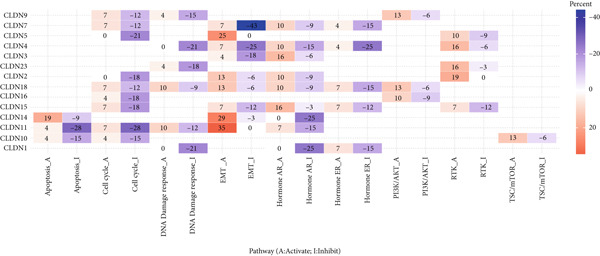
(g)

### 3.2. Two Distinct Clusters of CLDN Family

Firstly, we analyzed the clustering capabilities of the CLDN family, and Figure 2a visualized the global situation. Using the ConsensusClusterPlus package, we determined the ideal cluster number. It was found that *k* = 2, with the flattest CDF curve, is the optimal choice (Figure 2b). Figure 2c presents the delta area plot used to determine the optimal number of clusters. The substantial decrease in delta area from *k* = 2 to *k* = 3, followed by a flattening curve, supports our selection of *k* = 2 as the optimal clustering parameter. Subsequently we evaluated the clustering tendency by PCA. CLDN clusters were separated significantly, indicating a high‐quality consensus cluster result (Figure 2d). Then, we explored the OS of glioma patients in Cluster 1 and Cluster 2, *p* < 0.001. K‐M curves showed that Cluster 1 had higher and more prolonged survival than Cluster 2 (Figure 2e). Figure 2f demonstrated significant differences in immune‐related signatures between Cluster 1 and Cluster 2. Moreover, Figure 2g,h showed the respective global view of mutational distribution in Cluster 1 and Cluster 2. As a biomarker related to the malignancy of gliomas, IDH1 mutation took up 65% of the general in Cluster 1, higher than that of Cluster 2, 54%. Cellular tumor antigen p53 (TP53) alteration was presented similarly in Cluster 1 (45%) and Cluster 2 (39%). In Cluster 1, the following three genes ranked by frequency were ATRX (29%), CIC (18%), and TTN (15%), while those in Cluster 2 were ATRX (22%), EGFR (14%), and TTN (14%).

Figure 2(a) Consensus matrix for *k* = 2, showing the clustering of samples into two distinct groups. (b) Consensus CDF plot for determining the optimal cluster number. (c) Delta area plot indicating the relative change in the area under the CDF curve for different *k* values. (d) PCA plot illustrating the separation between Cluster 1 and Cluster 2. (e) Kaplan–Meier survival curves comparing overall survival between Cluster 1 and Cluster 2. (f) Box plot showing immune scores for the two clusters. (g, h) Oncoplots depicting the mutation profiles of the Top 20 most frequently mutated genes in Cluster 1 and Cluster 2, respectively.

**Figure:**
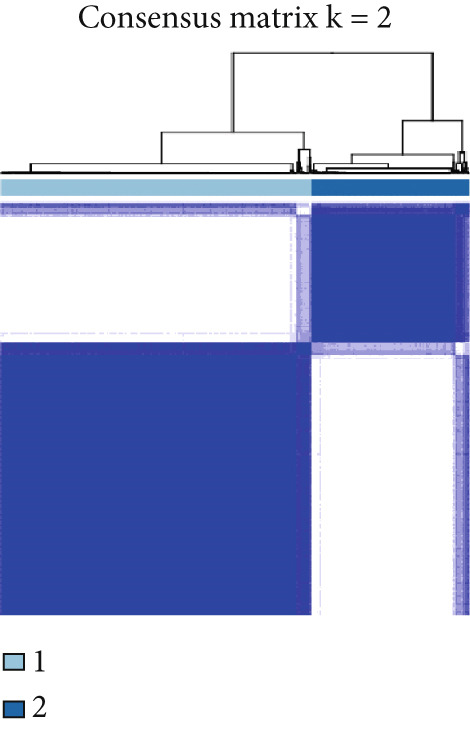
(a)

**Figure:**
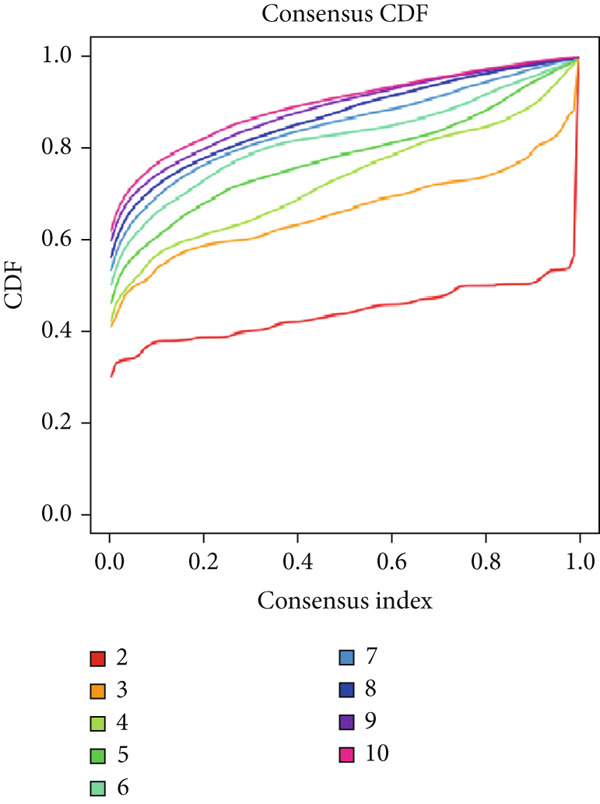
(b)

**Figure:**
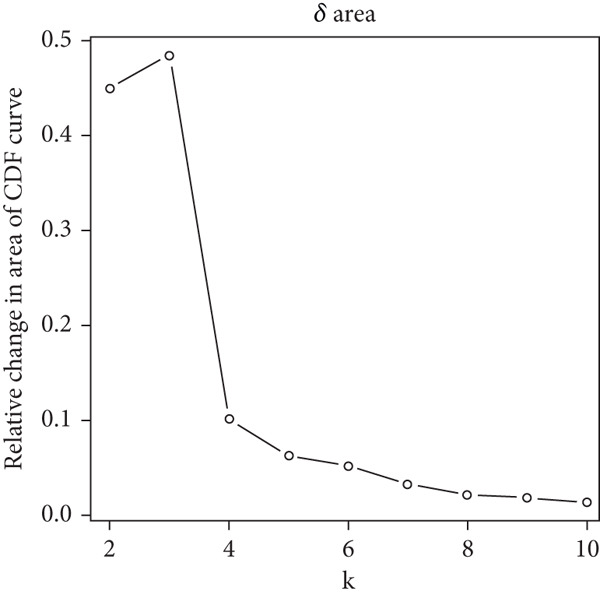
(c)

**Figure:**
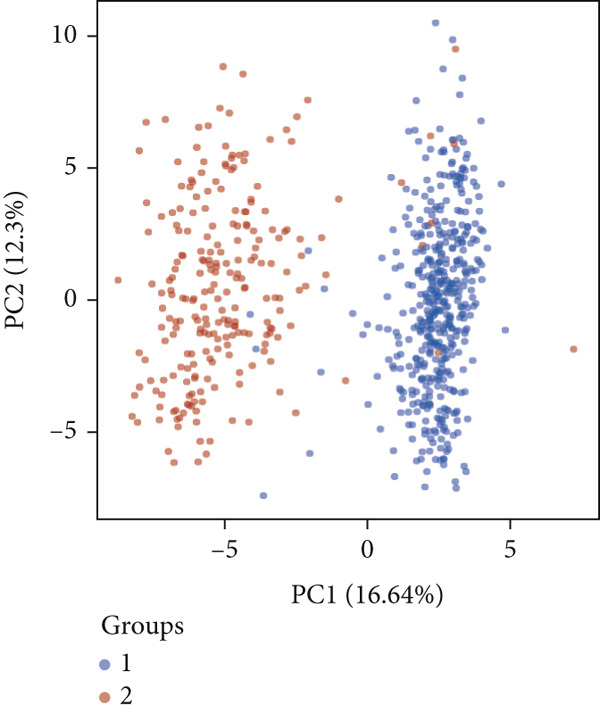
(d)

**Figure:**
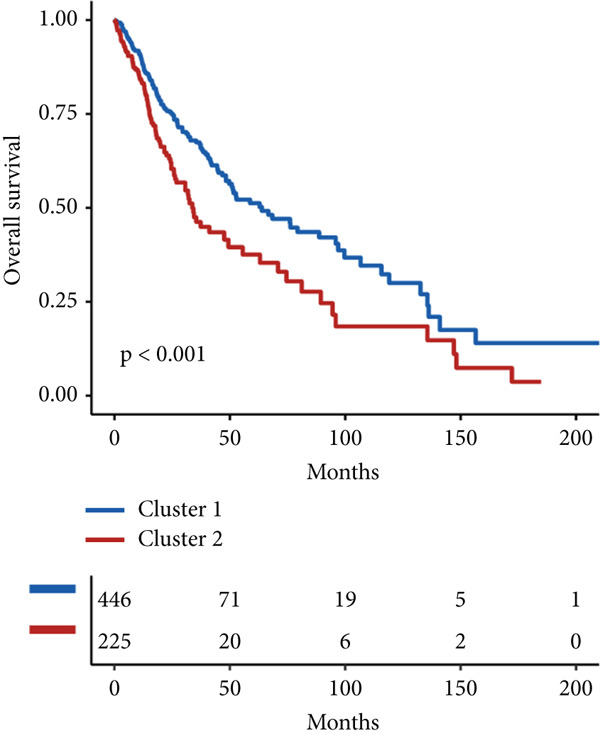
(e)

**Figure:**
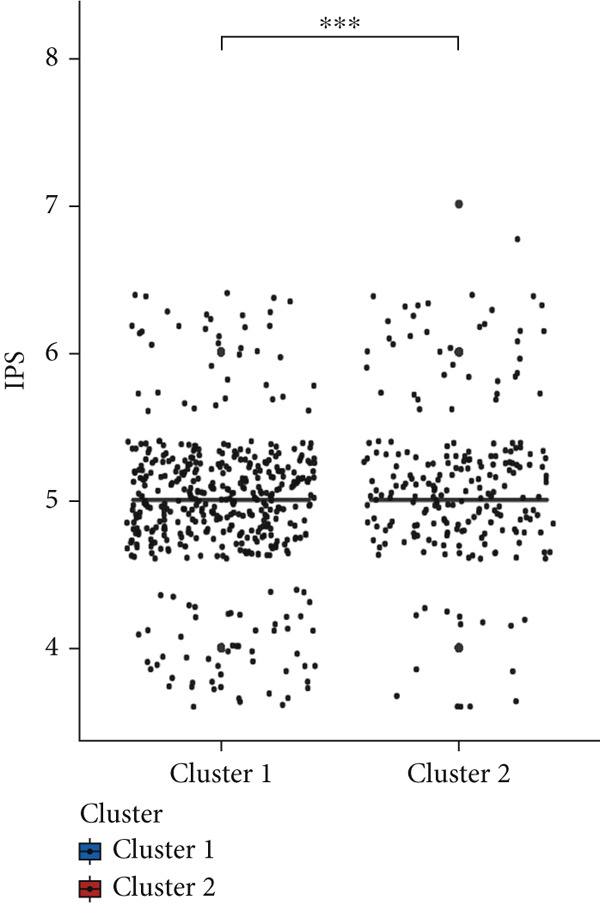
(f)

**Figure:**
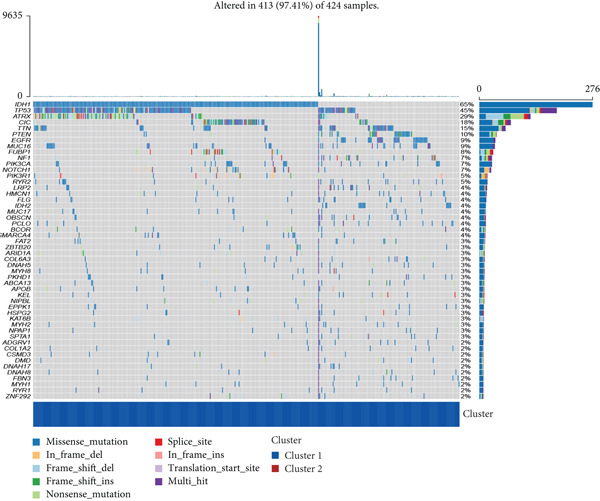
(g)

**Figure:**
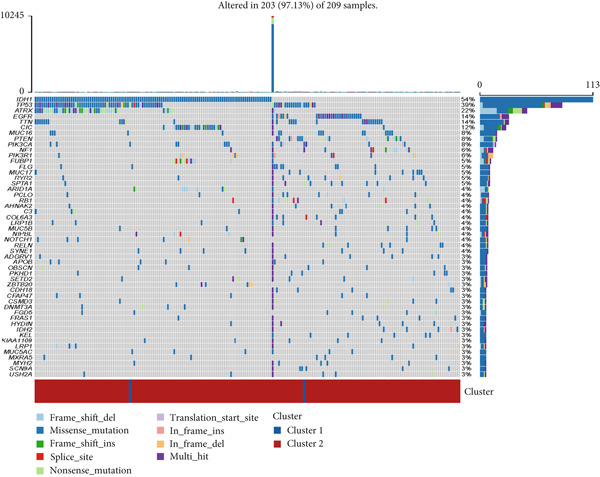
(h)

### 3.3. Distinct Genomic Profiles of the Two Clusters

We observed significant differences in OS and immune characteristics between Cluster 1 and Cluster 2, leading us to hypothesize distinct genomic profiles for these clusters. We analyzed the co‐occurrence and mutual exclusivity of the 25 most frequently altered genes in Cluster 1 (Figure 3a) and Cluster 2 (Figure 3b). In both clusters, the strongest co‐occurring gene mutation pairs were IDH1 with ATRX, IDH1 with CIC, IDH1 with NOTCH1, and ATRX with TP53. Conversely, IDH1 and EGFR were identified as mutually exclusive pairs across the clusters. Higher co‐occurrence typically indicates functional linkage to glioma proliferation. Furthermore, a forest plot (Figure 3c) illustrates the 17 genes with the most significant variation between the clusters. Apart from IDH1 and MYH8, the other nine genes are more prone to mutations in Cluster 2. Additionally, we compared the frequency of various somatic mutations between the clusters, including single‐nucleotide polymorphism (SNP), single‐nucleotide variant (SNV), deletion, insertion, and intergenic region (IGR). The frequencies of insertions and deletions did not show statistical differences, whereas SNPs were slightly more prevalent in Cluster 2 (Figure 3d). Among the identified SNVs, C to T transitions were more common, particularly in Cluster 2, where they were the most frequent mutation (Figure 3e). Moreover, alterations in splice regions and missense mutations were more prevalent in Cluster 2 compared to Cluster 1 (Figure 3f). Figure 3g illustrates the chromosomal alterations observed across the two clusters. The heatmap reveals distinct patterns of chromosomal gains and losses between clusters, with Cluster 1 showing more frequent alterations in chromosomes.

Figure 3(a, b) Heatmaps showing the co‐occurrence and mutual exclusivity of the top 25 most altered genes in Cluster 1 and Cluster 2. (c) Forest plot comparing the log odds ratio of gene alterations between Cluster 2 and Cluster 1. (d) Box plots displaying the distribution of variant types (SNP and DEL) between the clusters. (e) Box plots of single‐nucleotide variants (SNVs) categorized by nucleotide changes in each cluster. (f) Box plots presenting variant classifications, such as splice site and missense mutations, across the clusters. (g) Chromosomal plots illustrating the frequency of copy number alterations in Cluster 1 and Cluster 2.

**Figure:**
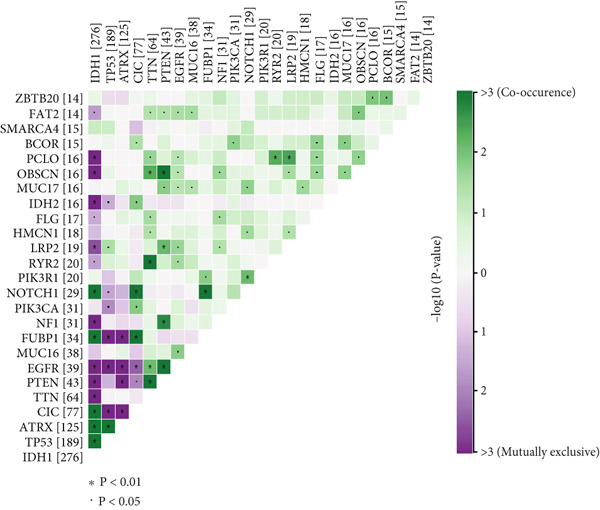
(a)

**Figure:**
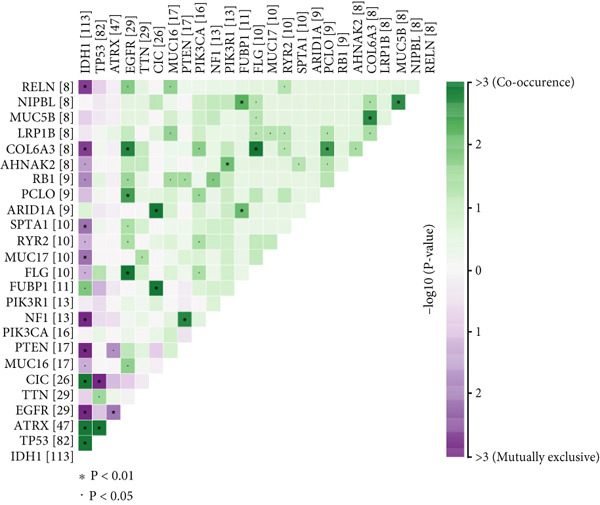
(b)

**Figure:**
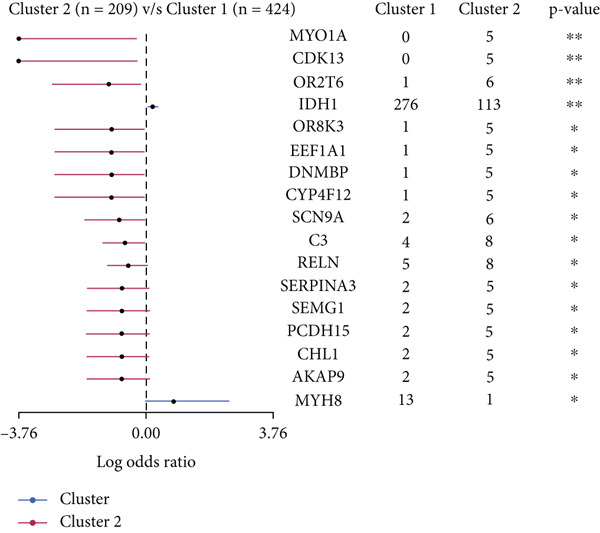
(c)

**Figure:**
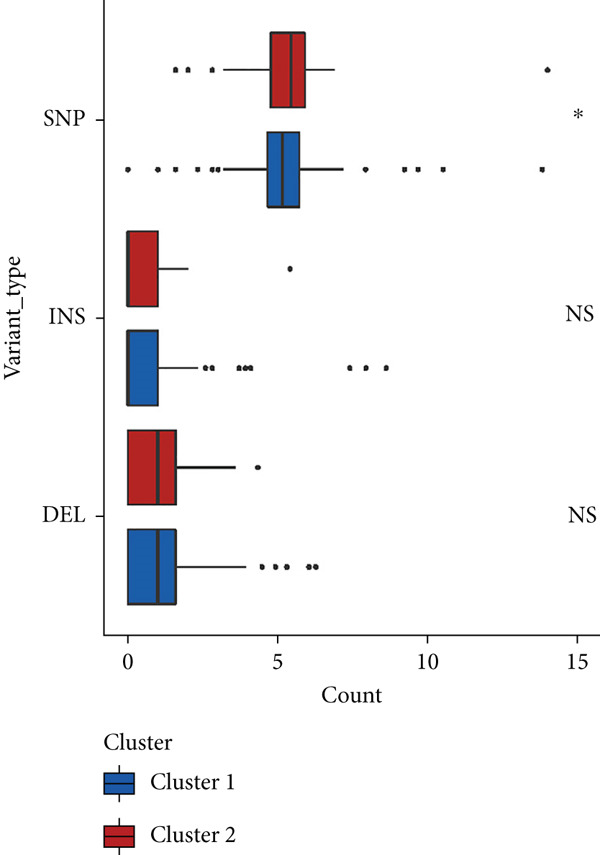
(d)

**Figure:**
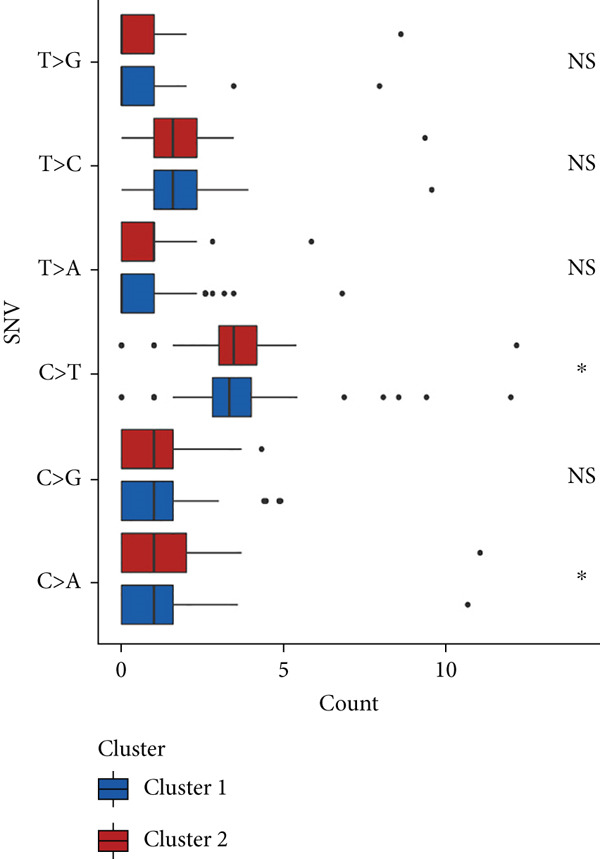
(e)

**Figure:**
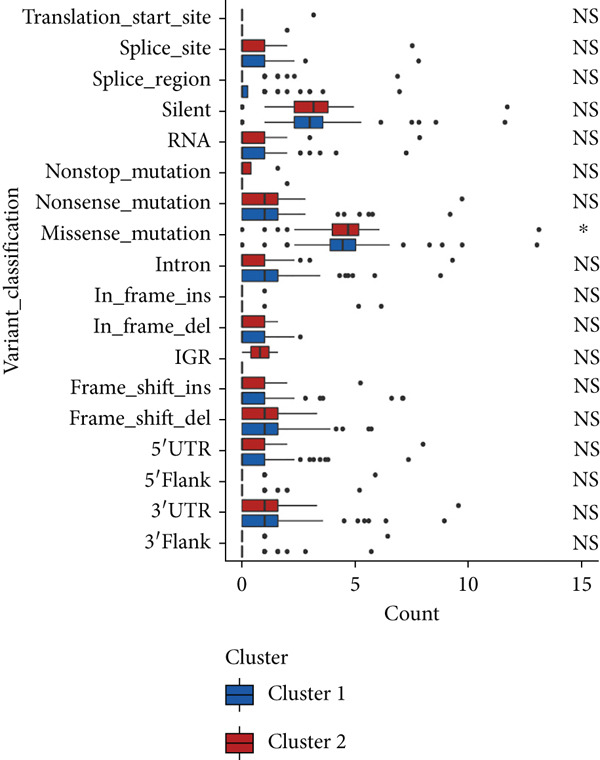
(f)

**Figure:**
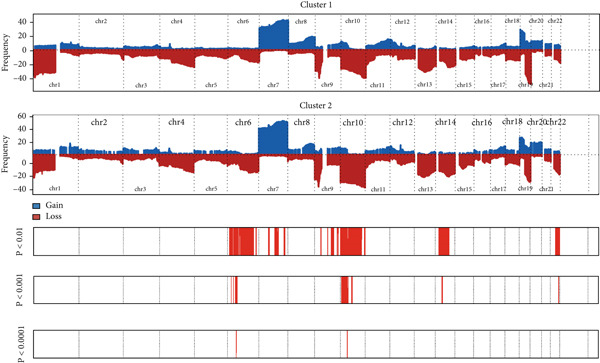
(g)

### 3.4. Immune Characteristics of Two Clusters

We investigated the TME characteristics of Cluster 1 and Cluster 2. We evaluated the ESTIMATE, Immune, and Stromal Scores of the two clusters in the TCGA dataset (Figures 4a, 4b, and 4c). In the ESTIMATE Score and Immune Score evaluation, the scores of Cluster 2 were higher than those of Cluster 1. Meanwhile, Cluster 1 and Cluster 2 scores are demonstrated similarly in the Stromal Score evaluation. The two clusters could be thought to have a significant difference on account of *p* < 0.001. Besides, we calculated relating levels of 64 cell types by the xCell algorithm and clusters in TCGA (Figure 4d). Subtypes of glioma are defined into four groups: proneural (PN), classical (CL), neural (NE), and mesenchymal (ME), among which CL and ME are more severe. The analysis showed that some types of cells are different in the two clusters with statistical significance (Figure 4e). Plasma cells, CD4+ Tcm, neurons, and so forth, are more positively related to Cluster 1. Additionally, we used box plots to present the proportions of 22 TME cell types in tumor tissues with Cluster 1 and Cluster 2 (Figure 4f). Only four cell types had significant differences: Eosinophils, macrophages M2, T cells CD4 memory activated, and T cell gamma delta. Macrophages M2 in Cluster 2 are higher than in Cluster 1.

Figure 4(a–c) Box plots showing ESTIMATE, immune, and stromal scores, respectively, for both clusters, indicating differences in the tumor microenvironment. (d) Heatmap illustrating the immune cell infiltration patterns across clusters, with annotations for clinical and molecular features. (e) Heatmap displaying the expression levels of CLDN family genes in each cluster, alongside clinical attributes. (f) Box plots representing the proportions of various immune cell types in Cluster 1 and Cluster 2.

**Figure:**
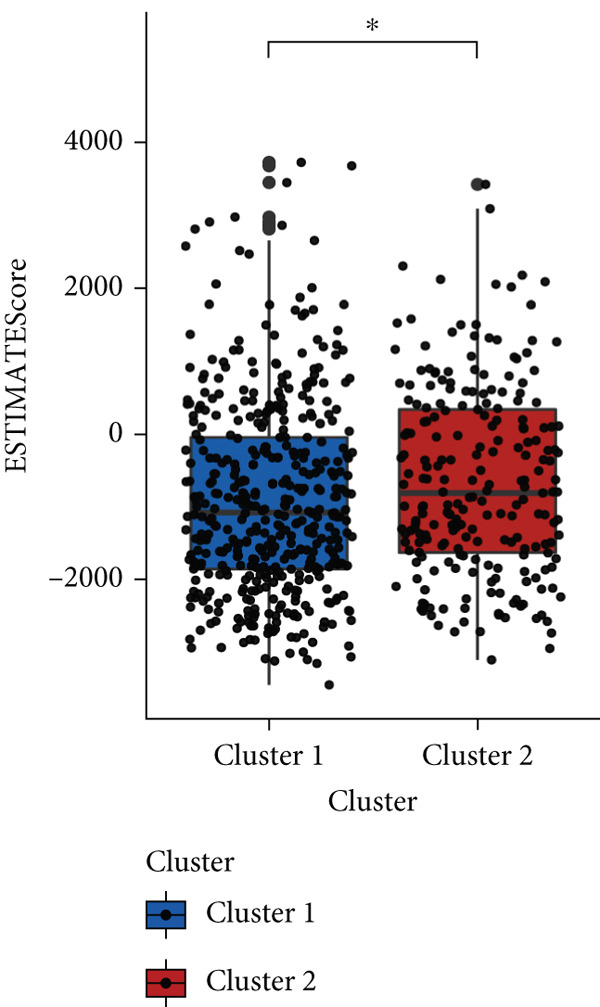
(a)

**Figure:**
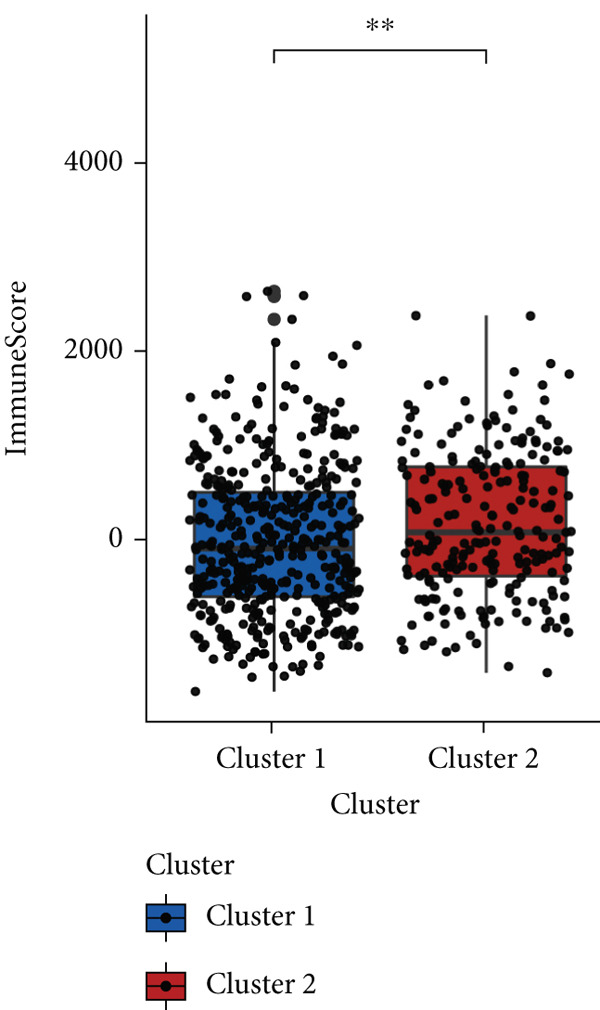
(b)

**Figure:**
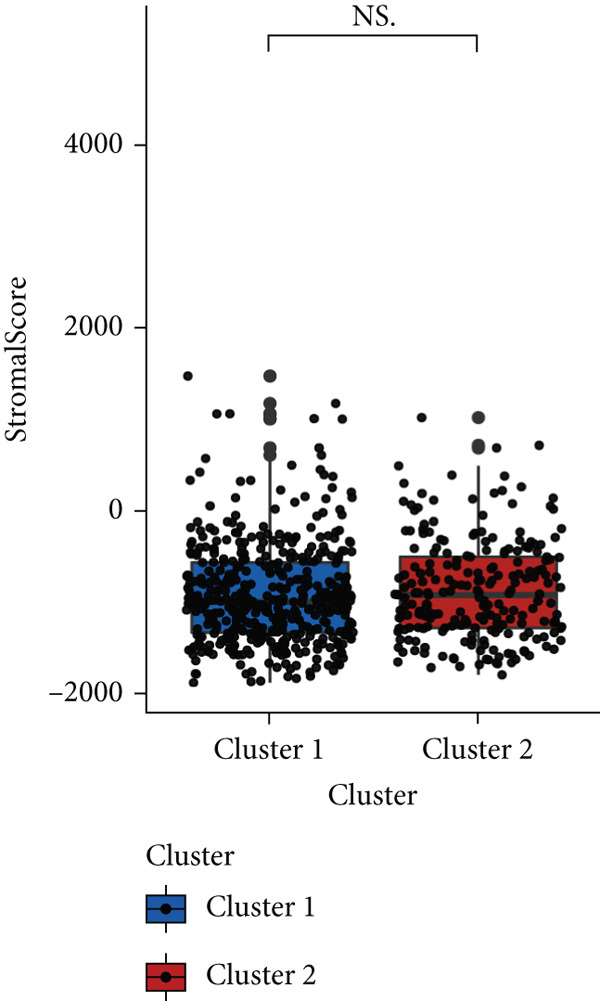
(c)

**Figure:**
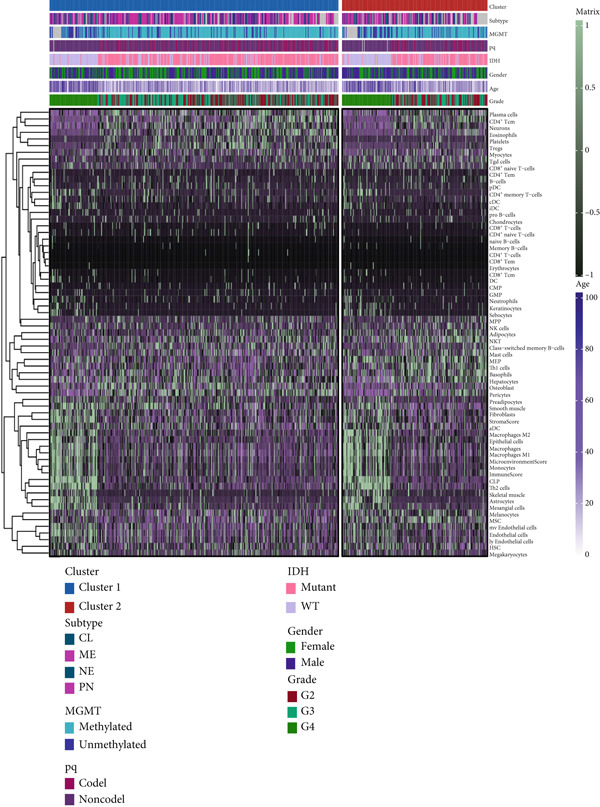
(d)

**Figure:**
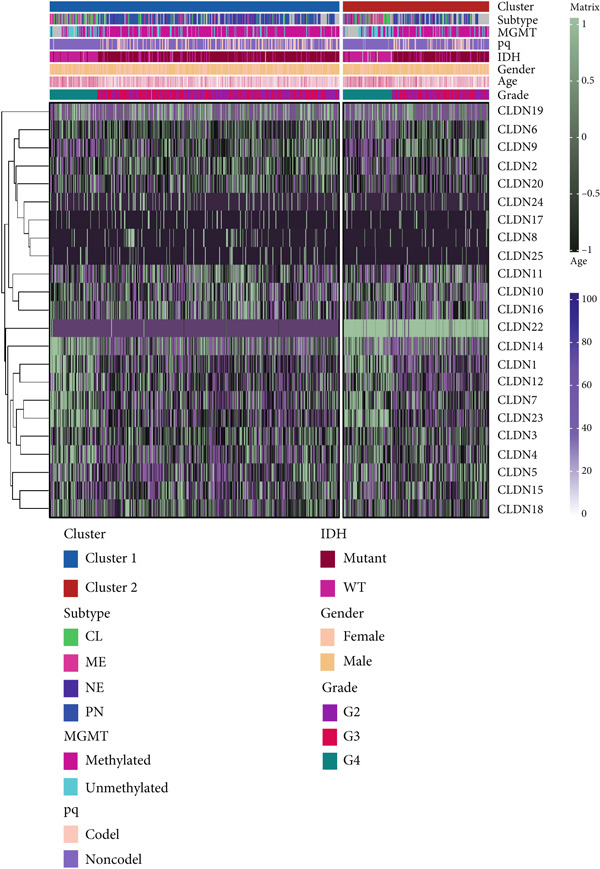
(e)

**Figure:**
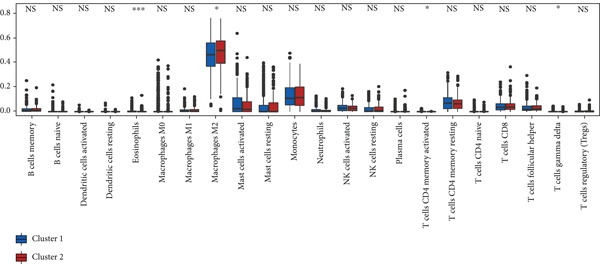
(f)

### 3.5. Identification of CLND22

To distinguish the two clusters more accurately and precisely, we executed machine learning and prediction on the two populations, screening out the most characteristic genes. Using LASSO‐LR, XGBoost, Boruta, PAMR, and RandomForest machine learning algorithms, we filtrated 20, 4, 9, 23, and 9 genes correspondingly (Figures 5a, 5b, 5c, 5d, and 5e). We used a Venn diagram to take the intersection of the five algorithms (Figure 5f). These two characteristic genes in the intersection corner had the most potential to classify the two clusters best. One of the selected genes was CLND22. Another was the sex‐determining region Y (SRY) determining male sex, so it was excluded. As a consequence, we identified CLDN22 as a biomarker of glioma prognosis.

Figure 5(a) LASSO regression plot showing the binomial deviance for different lambda values. (b) Feature importance plot from XGBoost analysis identifying key genes. (c) Boruta analysis results indicating confirmed important variables. (d) PAMR plot highlighting the threshold for gene selection in each cluster. (e) RandomForest analysis showing variable importance and accuracy. (f) Venn diagram illustrating the overlap of selected genes across different machine learning methods.

**Figure:**
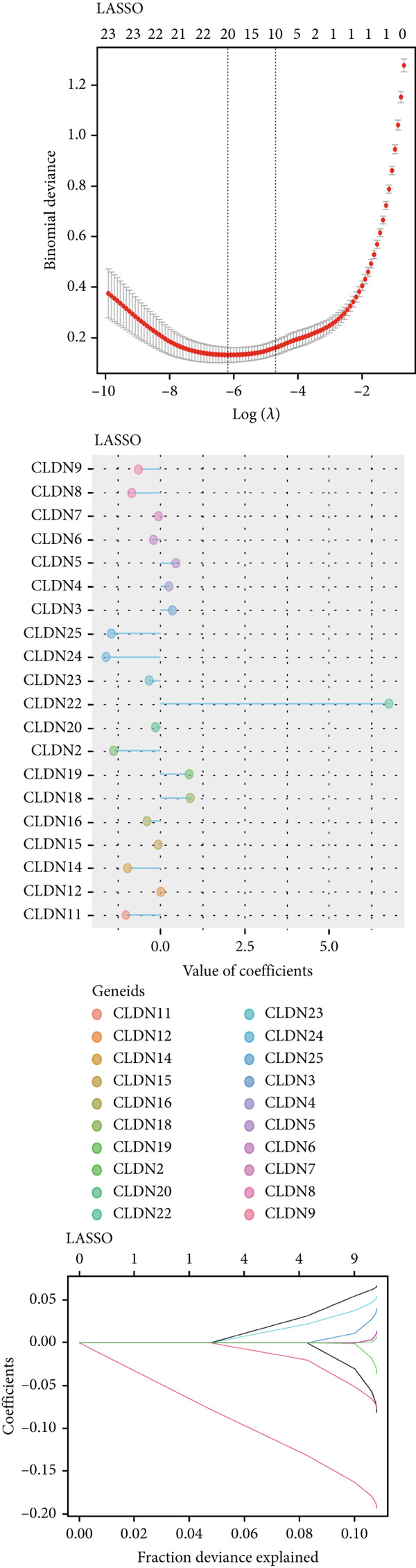
(a)

**Figure:**
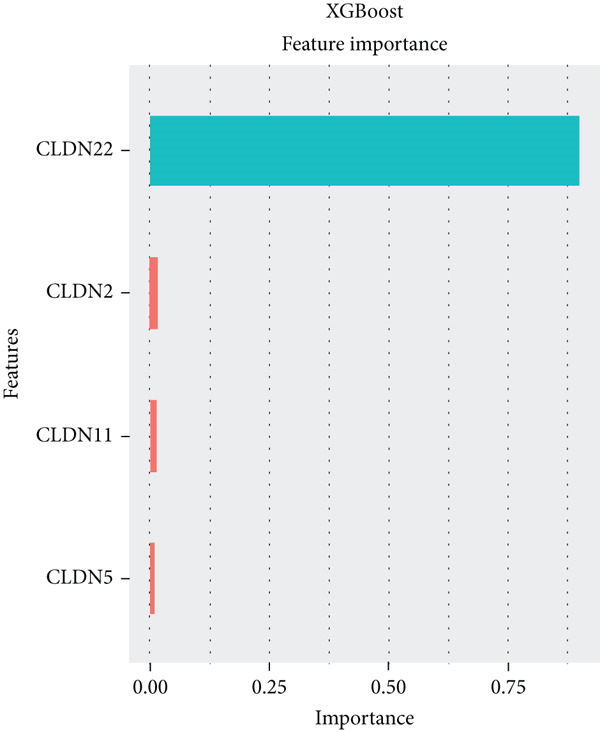
(b)

**Figure:**
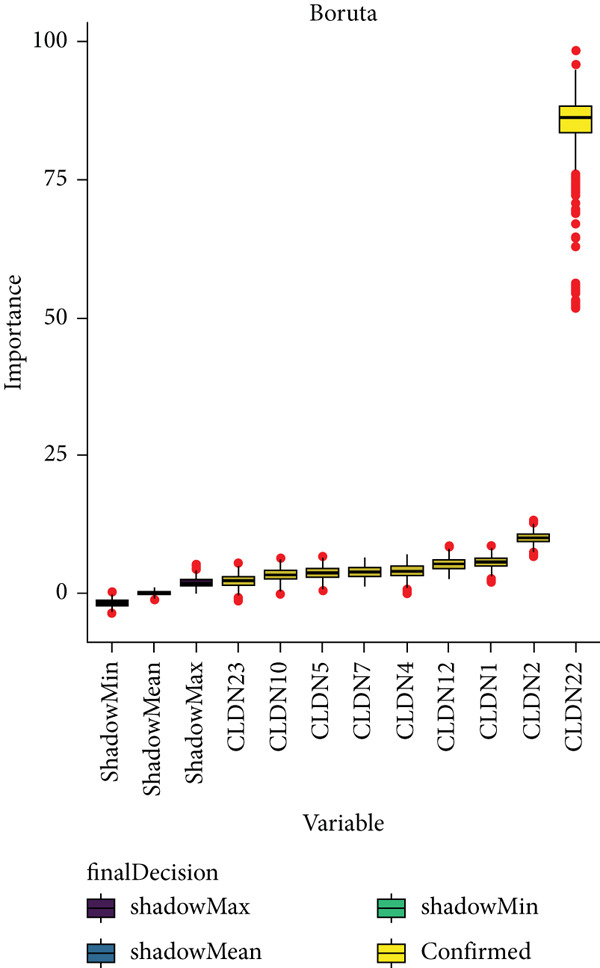
(c)

**Figure:**
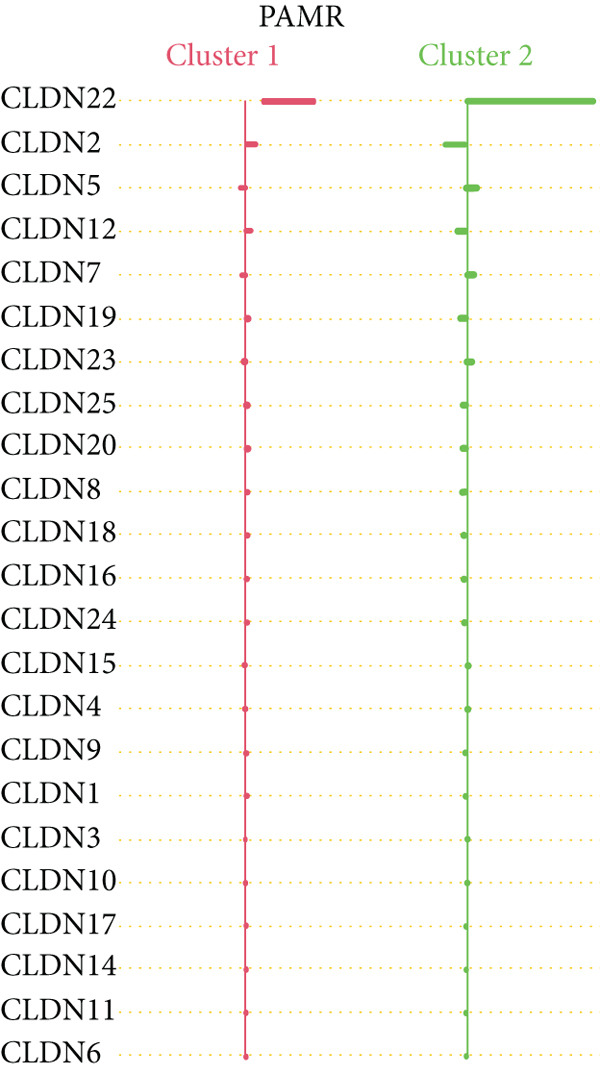
(d)

**Figure:**
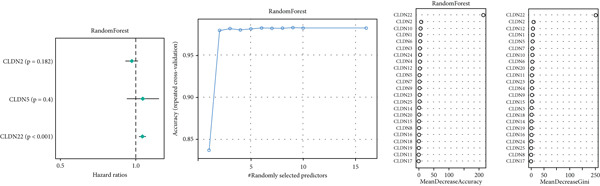
(e)

**Figure:**
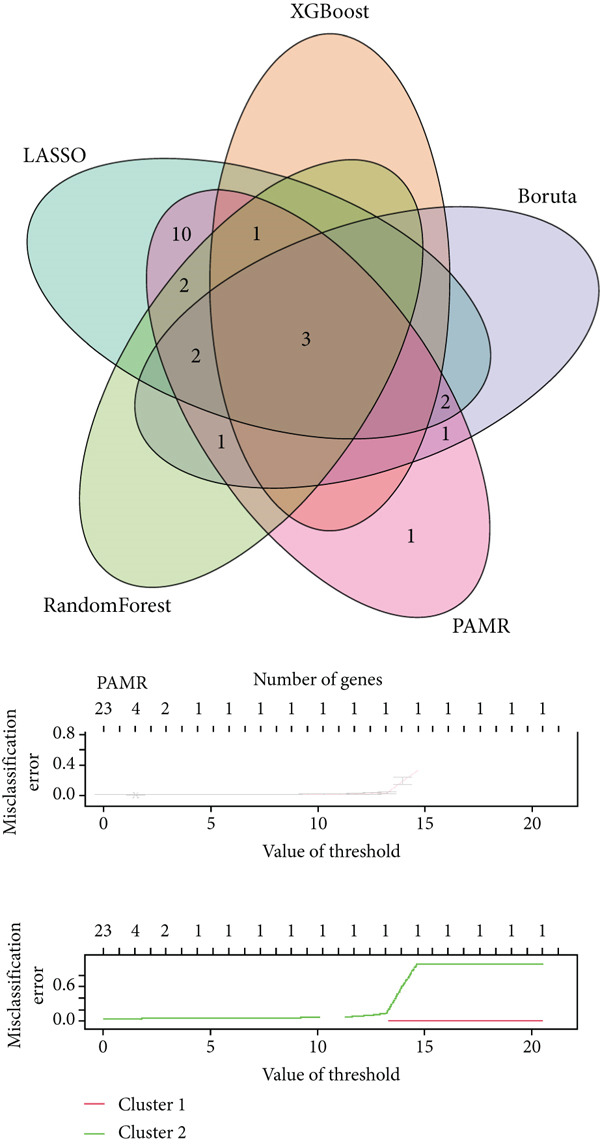
(f)

### 3.6. The Prognostic Potential of CLND22

**Figure 7:**
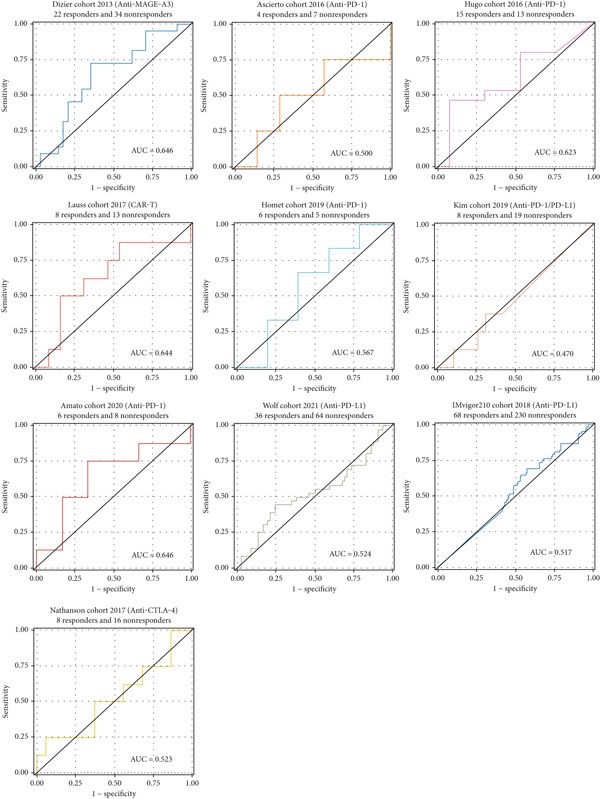
ROC curves for predicting immunotherapy response across various cohorts. Each subplot represents a different study cohort, assessing the predictive power of CLDN22 expression or other biomarkers for specific immunotherapies.

The survival analysis of different CLND22 expressions in pan‐glioma, LGG, and GBM was performed based on the TCGA datasets (Figure 6a). K‐M curves demonstrated significantly worse survival outcomes in glioma patients with low CLDN22. Consistently, time‐dependent ROC curves of 1‐year, 3‐year, and 5‐year OS in the TCGA dataset (AUC values, 0.568, 0.588, and 0.615, respectively) confirmed the prognostic value of the CLDN22 (Figure 6b). Besides, CLDN22 showed considerable capacity as an independent prognostic factor with IDH, 1p19q, MGMT, and subtype based on univariate and multivariate Cox regression analysis (Figure 6c). Figure 6d reveals that high CLDN22 expression was predominantly associated with higher tumor grade (G4), the ME subtype, IDH wild‐type status, and unmethylated MGMT promoter, suggesting its potential role as a molecular marker for tumor aggressiveness and specific glioma subtypes. Besides, Figure 6e demonstrated the abundance of infiltrating immune cell groups with divergent CLDN22 expressions identified by the CIBERSORT, the ESTIMATE, the MCP, and the TIMER algorithms of TCGA datasets. With the increasing expression of CLDN22, the proportion of cytotoxic lymphocytes, neutrophils, and endothelial cells decreased. According to the immunotherapy response analysis, CLDN22 achieved an AUC of more than 0.5 in 8 of the 25 immunotherapy cohorts. As shown in Supporting Information 1: Figure S1, the predictive performance of CLDN22 was benchmarked against multiple established biomarkers (e.g., TIDE, MSI score, TMB, and CD274), and CLDN22 demonstrated comparable or superior predictive value in certain cohorts, suggesting its potential as a candidate biomarker for immunotherapy response. In both LGG and HGG, patients with high CLDN22 expression exhibit significantly poorer OS compared with those with low CLDN22 expression (Supporting Information 2: Figure S2). We next assessed the predictive value of CLDN22 across 10 immunotherapy cohorts using ROC analysis (Figure 7). CLDN22 showed notable predictive power in the Dizier cohort 2013 (anti‐MAGE‐A3, AUC = 0.646), Lauss cohort 2017 (CAR‐T, AUC = 0.644), and Amato cohort 2020 (anti‐PD‐1, AUC = 0.646), while moderate prediction was observed in the Hugo 2016 (AUC = 0.623) and Homet 2019 (AUC = 0.567) cohorts. In contrast, CLDN22 showed limited predictive value in the Acsierto 2016 (AUC = 0.500), Kim 2019 (AUC = 0.470), Wolf 2021 (AUC = 0.524), IMvigor210 2018 (AUC = 0.517), and Nathanson 2017 (AUC = 0.523) cohorts. Collectively, these data demonstrate that CLDN22 exhibits variable but promising predictive ability under certain immunotherapy contexts, particularly in anti‐MAGE‐A3, CAR‐T, and anti‐PD‐1 therapies.

Figure 6(a) Kaplan–Meier survival curves for patients with high and low CLDN22 expression in different datasets. (b) ROC curves for overall survival prediction using CLDN22 expression. (c) Forest plot showing hazard ratios from univariate and multivariate Cox regression analyses. (d) Heatmap of CLDN22 expression and associated clinical features. (e) Heatmap displaying the immune cell infiltration levels in relation to CLDN22 expression.

**Figure:**
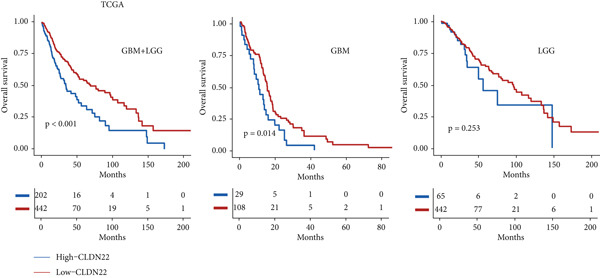
(a)

**Figure:**
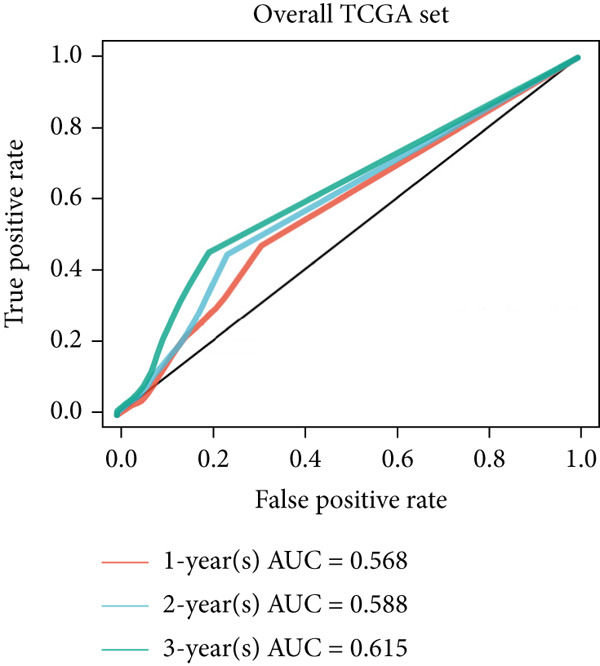
(b)

**Figure:**
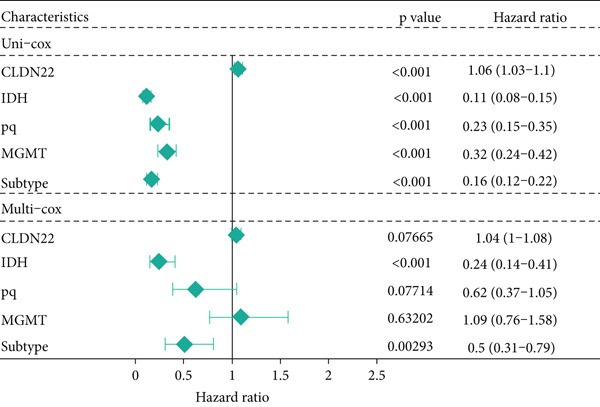
(c)

**Figure:**
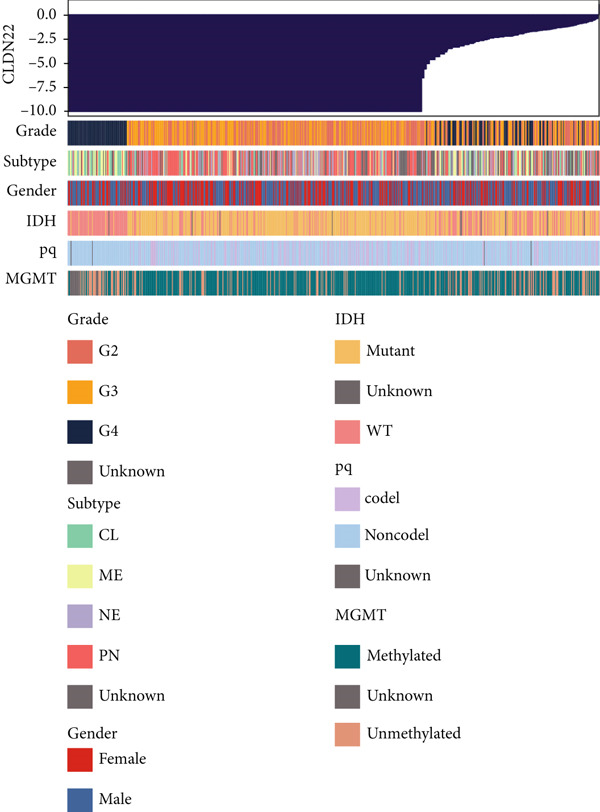
(d)

**Figure:**
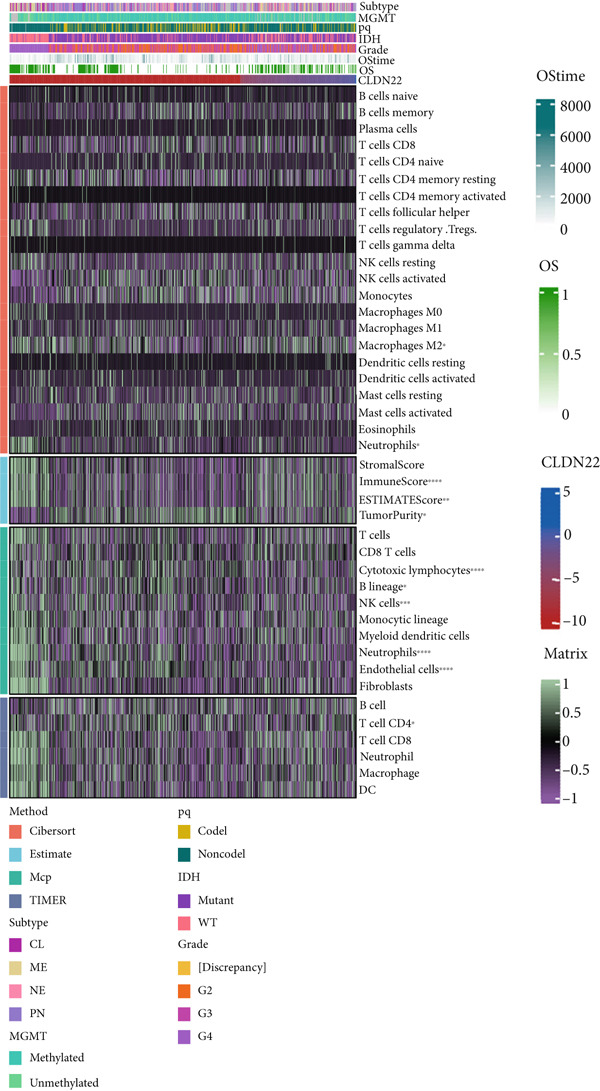
(e)

### 3.7. Drug Sensitivity Analysis

We further investigated the association between CLDN22 expression patterns and drug sensitivity across multiple datasets (Figure 8). The heatmaps illustrate distinct expression‐dependent drug responses. In the high‐expression group (Figure 8a), CLDN22 levels correlated positively with increased sensitivity to Mdivi‐1, MLN2238, and ML210, whereas resistance trends were observed for docetaxel and dasatinib (Figure 8c). Conversely, the low‐expression group (Figure 8b,d) exhibited enhanced sensitivity to SB225002 and gefitinib but reduced sensitivity to compounds such as PD‐168393 and phthalylsulfacetamide. These findings highlight differential drug response patterns stratified by CLDN22 expression, underscoring the potential to tailor treatment strategies based on expression profiles.

Figure 8(a) Correlations for high CLDN22 expression indicating increased sensitivity to certain compounds. (b) Correlations for low CLDN22 expression showing sensitivity profiles. (c) Drugs with enhanced sensitivity in high‐expression groups. (d) Drugs with increased sensitivity in low‐expression groups. Color intensity represents the strength and direction of correlation, with red indicating positive correlation and blue indicating negative correlation.

**Figure:**
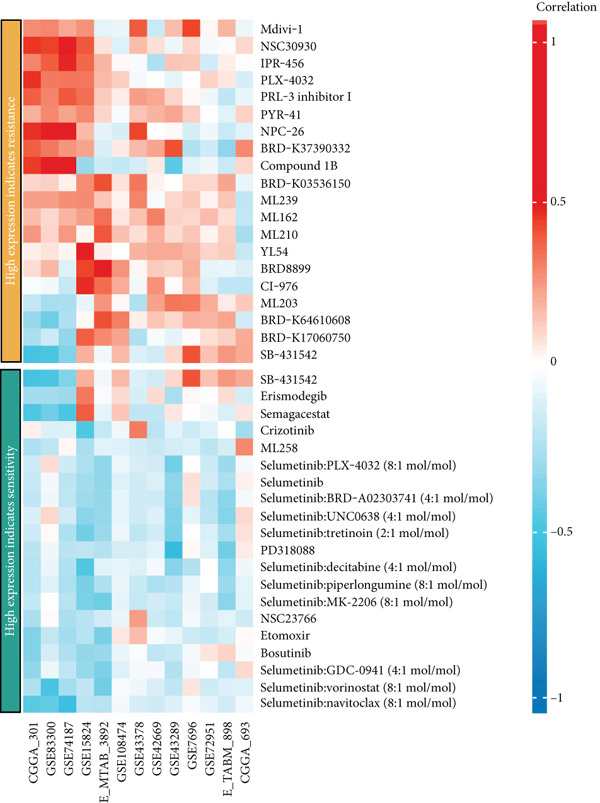
(a)

**Figure:**
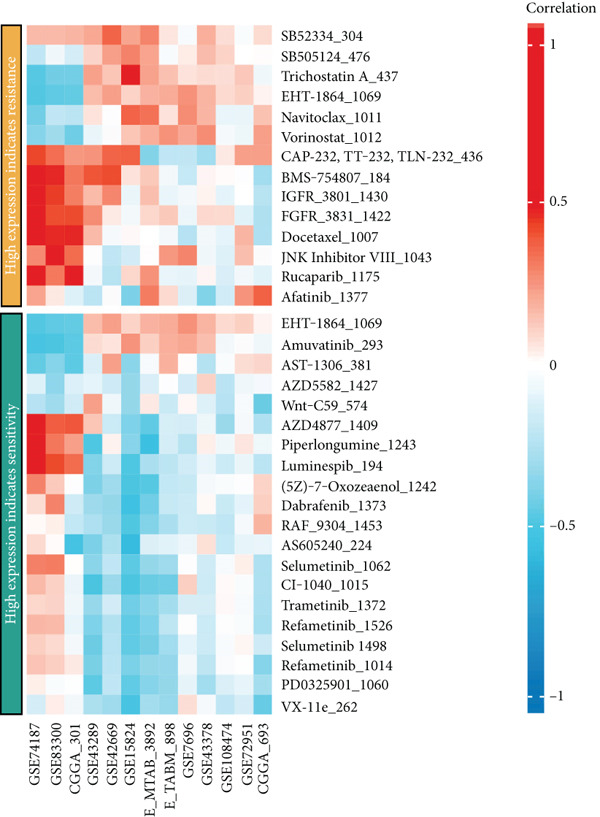
(b)

**Figure:**
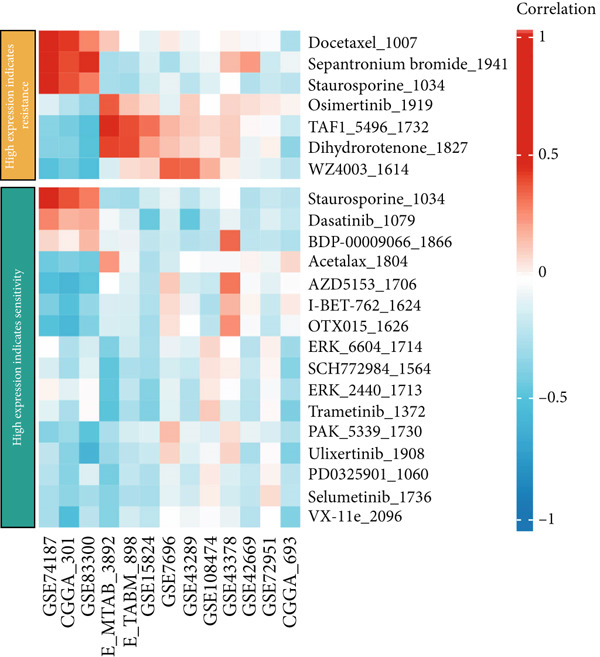
(c)

**Figure:**
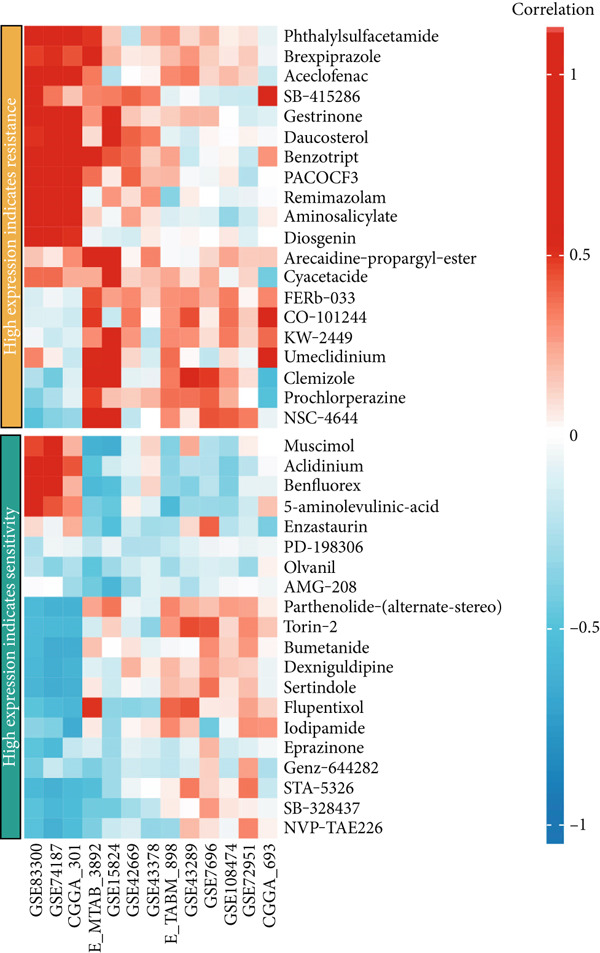
(d)

### 3.8. GO and KEGG Enrichment Analyses′ Results

Using the DAVID database, we performed GO enrichment analysis for LGG and GBM separately. For LGG, the top enriched BP terms included cellular aromatic compound metabolic process, heterocycle metabolic process, and nucleic acid metabolic process. CC terms were mainly associated with intracellular structures and organelles, while MF terms highlighted nucleic acid binding and DNA binding activities (Figure 9a). For GBM, enriched BP terms were generally similar, encompassing nitrogen compound metabolic process, cellular macromolecule metabolic process, and aromatic compound metabolic process. CC terms focused on intracellular compartments including the nucleus, while MF terms showed broad enrichment in nucleic acid binding and heterocyclic compound binding (Figure 9b). Although overlap exists, compared with LGG, GBM enrichment emphasized more global macromolecule and nitrogen metabolism processes, whereas LGG was more biased toward aromatic/nucleobase metabolic regulation, suggesting that CLDN22 may influence subtype‐specific metabolic and transcriptional programs related to glioma progression (Supporting Information 3: Table S1).

Figure 9GO enrichment analysis of CLDN family genes. (a) Bar plots for detected and enriched genes associated with biological processes, cellular components, and molecular functions inLGG. (b) Similar analysis for GBM, highlighting the number of genes involved in each GO term. Categories include sensory perception, nucleic acid processes, and membrane components.

**Figure:**
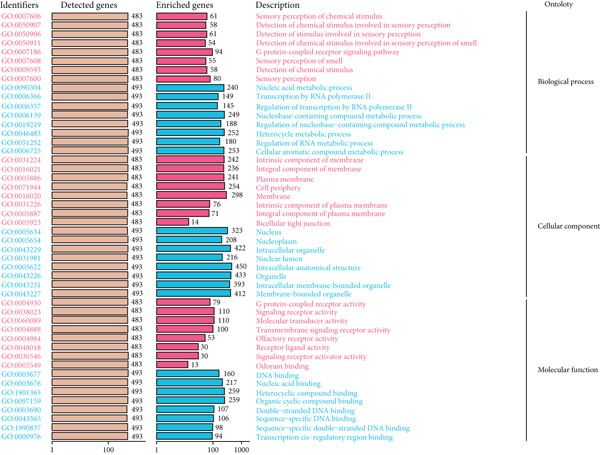
(a)

**Figure:**
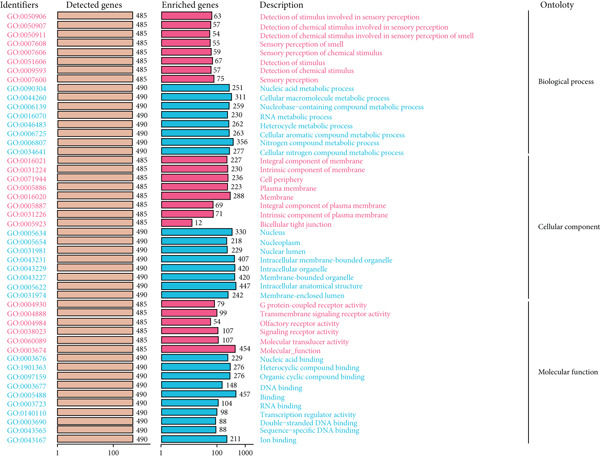
(b)

In KEGG analysis, LGG enrichment was observed in pathways such as olfactory transduction, cell adhesion molecules, leukocyte transendothelial migration, cytokine–cytokine receptor interaction, hepatitis C, and tight junction (Figure 10a). In contrast, GBM enrichment included not only partially overlapping pathways (olfactory transduction, cell adhesion molecules, tight junction, hepatitis C, and leukocyte transendothelial migration) but also unique signaling‐related pathways such as Wnt signaling and neuroactive ligand–receptor interaction (Figure 10b). These findings together indicate that CLDN22 is linked to both shared and distinct BPs in LGG and GBM, potentially driving subtype‐specific mechanisms of glioma growth and therapeutic response (Supporting Information 4: Table S2).

Figure 10KEGG pathway enrichment analysis of CLDN family genes. (a) Bar plots showing the number of detected and enriched genes across various pathways, categorized into levels of cellular processes, environmental information processing, genetic information processing, human diseases, metabolism, and organismal systems in LGG. (b) Similar analysis for GBM, highlighting pathways such as cell cycle, DNA replication, and metabolic processes.

**Figure:**
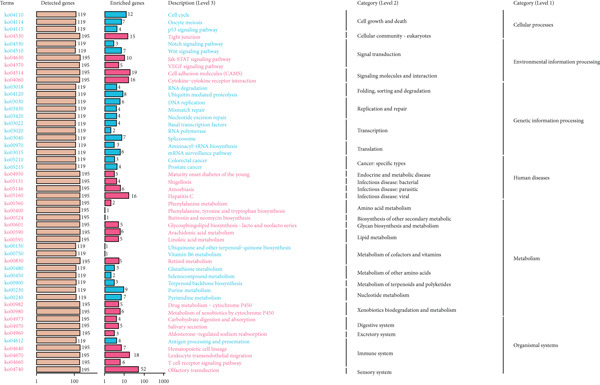
(a)

**Figure:**
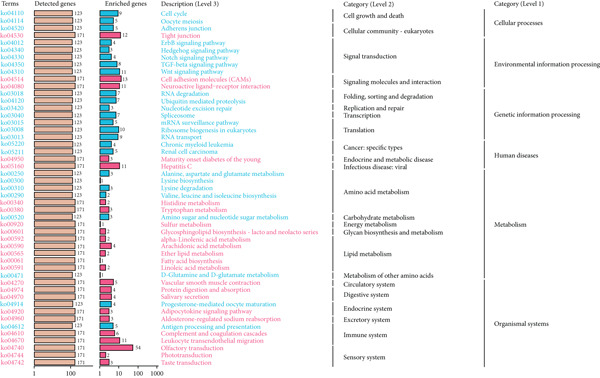
(b)

## 4. Discussion

Tight junctions are an essential component of intercellular junctions; they maintain cell polarity, permeability, and adhesion and regulate cell proliferation and differentiation [24]. The CLDN family is an indispensable part of TJs. Tumor progression is characterized by cancer cell migration, invasion, and metastasis [25]. CLDNs are thought to play a critical role in these processes, as their loss leads to the disruption of cell adhesion in a tissue‐dependent manner [26]. Despite extensive studies on the CLDN family in cancers, most research has focused on liver, ovarian, endometrial, and esophageal cancers, while glioma studies remain relatively limited [27–30]. We systematically explored the role of CLDN family genes in glioma prognosis, immune microenvironment, and therapeutic response prediction. Our results revealed significant heterogeneity in the expression, genetic alterations, and functional roles of CLDNs across different cancer types and patient subgroups. CLDN22 emerged as a critical biomarker associated with survival outcomes, TME characteristics, and therapeutic response.

The comprehensive analysis of CLDN family genes in gliomas revealed complex regulatory mechanisms involving CNVs and DNA methylation. Our findings demonstrated significant positive correlations between CNV and mRNA expression for several CLDN genes, particularly CLDN12 and CLDN15, in both GBM and LGG cohorts. This linear relationship suggests that these CLDN family members may be primarily regulated through copy number alterations rather than complex transcriptional or epigenetic mechanisms. Given the crucial roles of CLDN12 and CLDN15 in maintaining tight junctions and barrier function, their CNV‐driven expression changes might significantly impact glioma progression through altered cell–cell adhesion and blood–brain barrier integrity [31]. DNA methylation emerged as another crucial regulatory mechanism, with CLDN3 and CLDN11 showing notable negative correlations between methylation levels and gene expression, especially in LGG. This inverse relationship reflects the canonical mechanism where DNA methylation, especially in CpG‐rich promoter regions, leads to transcriptional silencing through chromatin remodeling and reduced transcription factor accessibility [32]. The more prominent manifestation of this relationship in LGG suggests a grade‐specific epigenetic regulation pattern, possibly reflecting different stages of tumor progression. Their methylation‐mediated suppression might contribute to tumor progression through altered cell–cell adhesion and increased invasive potential.

After dividing into two distinct clusters, we found that Cluster 1 demonstrated significantly longer survival than Cluster 2. The observed differences in mutation patterns between Cluster 1 and Cluster 2 reflect the two subtypes′ distinct biological characteristics and genomic instability. IDH1 mutations, which frequently co‐occur with ATRX, TP53, and NOTCH1 mutations in both clusters, highlight its critical role in glioma progression through metabolic reprogramming. ATRX, TP53, and NOTCH1 play critical roles in the initiation and progression of gliomas. These three genes collectively influence tumor biology through the regulation of chromatin remodeling, telomere maintenance, cell cycle control, apoptosis, tumor stem cell characteristics, and angiogenesis [33–35]. ATRX mutations frequently co‐occur with TP53 mutations, promoting tumor proliferation by activating the alternative lengthening of telomeres mechanism, and are closely associated with IDH‐mutant gliomas [36]. The loss of TP53 function leads to cell cycle dysregulation, genomic instability, and resistance to chemotherapy. In contrast, NOTCH1 enhances tumor invasiveness and therapeutic resistance by maintaining tumor stem cell properties and promoting angiogenesis [37]. The mutual exclusivity of IDH1 and EGFR mutations suggests divergent oncogenic pathways, with IDH1 mutations predominantly associated with LGGs and EGFR mutations linked to more aggressive glioblastomas [38]. The higher mutation frequency of MYH8 and other genes in Cluster 2 indicates increased genomic instability in this group, potentially contributing to its more aggressive phenotype. Furthermore, the higher prevalence of SNPs and the predominance of C → T transitions in Cluster 2 suggest that environmental stresses or epigenetic alterations, such as oxidative stress or DNA methylation, may influence this subtype. The increased frequency of splice region and missense mutations in Cluster 2 further supports its more complex molecular profile, potentially leading to disrupted RNA processing or altered protein function. Plasma cells, CD4+ central memory T cells, and neurons were more positively associated with Cluster 1, suggesting potentially better immune surveillance in this group [39, 40]. In contrast, macrophages M2 were significantly enriched in Cluster 2. M2‐like macrophages release immunosuppressive chemokines and cytokines to inhibit Th1‐type immune activity while enhancing Th2‐type immune responses. This activity reduces the control of inflammatory responses while simultaneously promoting tumor cell growth, drug resistance, angiogenesis, and tissue remodeling. Therefore, the M2‐driven immunosuppressive microenvironment in Cluster 2 contributes to its higher aggressiveness [41].

Machine learning has been widely used in disease marker exploration. Through machine learning approaches, we identified CLDN22 as a biological marker for glioma prognosis. Our findings reveal the multifaceted role of CLDN22 in glioma biology, prognosis, and immunotherapy response. High CLDN22 expression is significantly associated with poor survival outcomes and more aggressive tumor characteristics. Currently, the most successful clinical application of the CLDN family is CLDN18.2‐targeted CAR‐T therapy [27, 42]. According to recent research, approximately 20%–30% of gastric cancers show downregulation or loss of CLDN 18.2 expression [37]. The tumor immune microenvironment of CLDN 18.2‐positive gastric cancer is typically characterized by decreased NK cell counts and elevated neutrophil levels. Transcriptional analyses further reveal limited quantities of regulatory CD4+ T cells, CD8+ T cells, myeloid dendritic cells in the TME, and elevated B cell levels [43]. In this study, increasing CLDN22 expression was associated with decreased proportions of cytotoxic lymphocytes, neutrophils, and endothelial cells. CLDN22‐positive gliomas likely exhibit a “cold” immune phenotype, characterized by limited immune cell infiltration and an immunosuppressive TME. As a nonclassical CLDN family gene, CLDN22 has been relatively understudied in cancer. According to GeneCards (https://www.genecards.org/) annotations, CLDN22 plays a crucial role in the specific disappearance of tight junctions in intercellular spaces through calcium‐independent cell adhesion activity. Moreover, it functions in critical biological pathways, including blood‐brain barrier maintenance and immune cell migration [44]. CLDN22 maintains barrier function by regulating blood‐brain barrier permeability and potentially participates in immune cell migration and transendothelial processes through modulation of VCAM‐1/CD106 signaling. This mechanism explains how abnormal CLDN22 expression may regulate immune cell infiltration in the TME, leading to immune escape and enhanced tumor invasiveness. Regarding immunotherapy response prediction, CLDN22 demonstrated robust predictive capability in several immunotherapy cohorts, particularly for anti‐MAGE‐A3, CAR‐T, and anti‐PD‐1 treatments, outperforming traditional predictive indicators. This superior performance might be attributed to specific mechanisms that warrant further investigation. Subsequent GO and KEGG pathway analyses further confirmed CLDN22′s significant role in cell adhesion and tight junction signaling pathways in glioma.

Taken together, CLDN22 represents a promising biomarker for glioma prognosis, tumor aggressiveness, and immunotherapy response, warranting further investigation into its molecular mechanisms and clinical applications.

## 5. Limitation

However, our study is not without limitations. First, the lack of functional experimental validation restricts our ability to confirm the biological mechanisms underlying our findings. Second, the static nature of our data limits the exploration of dynamic changes in the relevant biomarkers over time. Lastly, the relatively small sample size impacts the precision and robustness of predictive models developed using machine learning. In future work, we aim to overcome these limitations by acquiring larger and more diverse datasets and conducting functional experiments. These efforts will enable a more comprehensive and in‐depth understanding of the regulatory roles of the identified genes.

**Nomenclature**
AUC: area under the curve
BP: biological process
CC: cellular component
CDF: cumulative distribution function
CGGA: Chinese Glioma Genome Atlas
CIC: Capicua transcriptional repressor
CLDN: claudin
CNV: copy number variation
EGFR: epidermal growth factor receptor
GBM: glioblastoma multiforme
GDSC: Genomics of Drug Sensitivity in Cancer
GO: Gene Ontology
IDH: isocitrate dehydrogenase
IGR: intergenic region
KEGG: Kyoto Encyclopedia of Genes and Genomes
KM: Kaplan–Meier
LASSO: least absolute shrinkage and selection operator
LGG: low‐grade glioma
MF: molecular function
MGMT: O6‐methylguanine‐DNA methyltransferase
OS: overall survival
PAMR: Prediction Analysis for Microarrays
PCA: principal component analysis
PD‐1: programmed cell death protein 1
ROC: receiver operating characteristic
SNP: single‐nucleotide polymorphism
SNV: single‐nucleotide variant
SRY: sex‐determining region Y
TCGA: The Cancer Genome Atlas
TMB: tumor mutation burden
TME: tumor microenvironment
TPM: transcripts per million

## Ethics Statement

The authors have nothing to report.

## Consent

The authors have nothing to report.

## Conflicts of Interest

The authors declare no conflicts of interest.

## Author Contributions

Hui Zheng and Jingsong Cheng contributed equally to this work.

## Funding

No funding was received for this manuscript.

## Supporting Information

Additional supporting information can be found online in the Supporting Information section.

## Supporting information


**Supporting Information 1** Figure S1: Receiver operating characteristic (ROC) curves for CLDN22 and other biomarkers across multiple immunotherapy cohorts, demonstrating predictive performance for treatment response. The AUC values are provided for each cohort and biomarker.

**Supporting Information 2** Figure S2: Kaplan–Meier survival curves for glioma patients with high and low CLDN22 expression levels in LGG and GBM cohorts, based on CGGA datasets. The survival analysis highlights the prognostic significance of CLDN22.

**Supporting Information 3** Table S1: Gene Ontology (GO) enrichment analysis results for CLDN22 in LGG and GBM cohorts, detailing the top enriched biological processes, molecular functions, and cellular components.

**Supporting Information 4** Table S2: Kyoto Encyclopedia of Genes and Genomes (KEGG) pathway enrichment analysis results for CLDN22 in LGG and GBM cohorts, identifying key pathways associated with glioma progression.

## Data Availability

The transcriptome data and clinical information of patients diagnosed with glioblastoma multiforme (GBM) and lower grade glioma (LGG) analyzed in this study are publicly available from The Cancer Genome Atlas (TCGA) database (https://www.cancer.gov/about-nci/organization/ccg/research/structural-genomics/tcga). All data used in this research can be accessed and downloaded from the TCGA portal.
