# Supplementary figures and images for: CLDN22 Serves as a Novel Prognostic Biomarker and Immunotherapy Response Predictor in Gliomas: A Comprehensive Multiomics Analysis
Source: Int J Genomics. 2025 Dec 20;2025:9367254. doi: 10.1155/ijog/9367254 (PMC12717531; doi:10.1155/ijog/9367254)

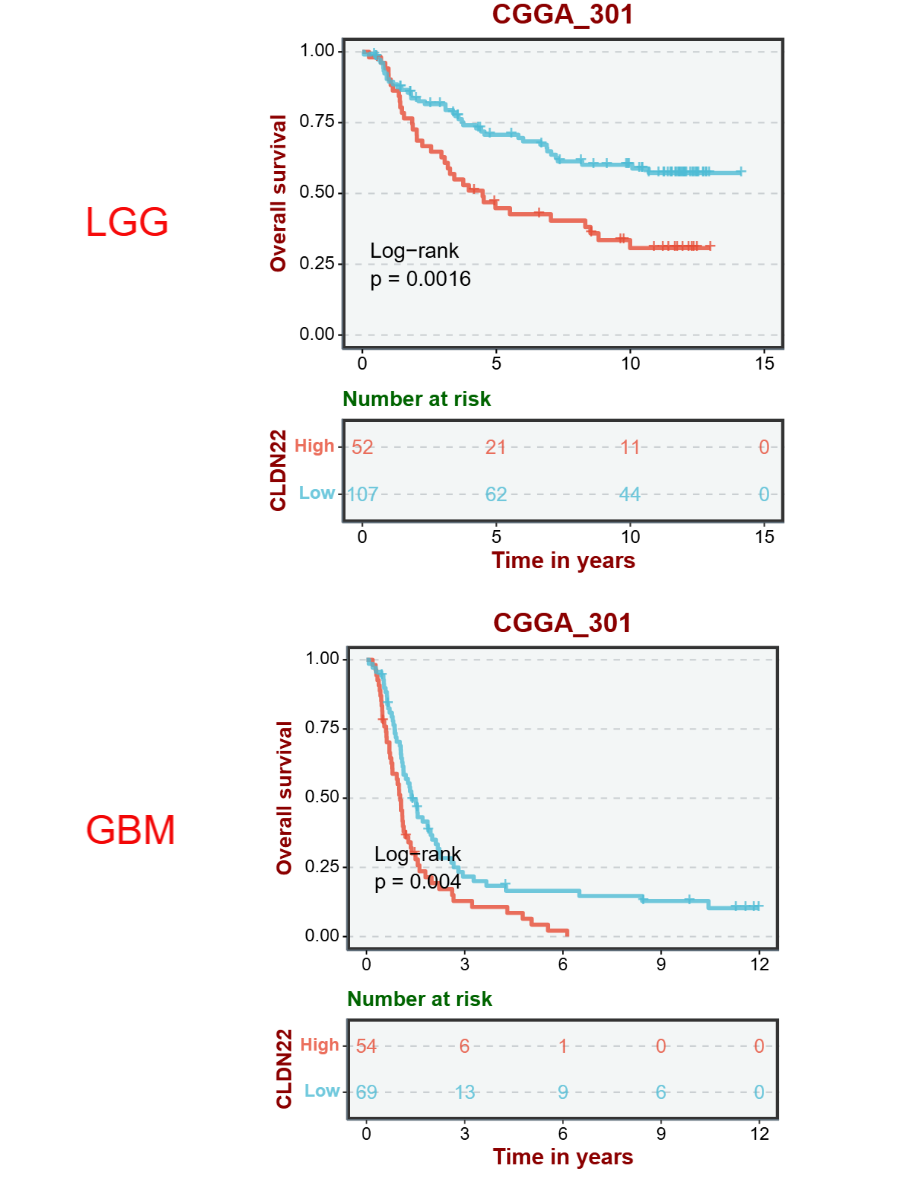

Supplement: Supplementary file 2 — Supporting Information 2 Figure S2: Kaplan–Meier survival curves for glioma patients with high and low CLDN22 expression levels in LGG and GBM cohorts, based on CGGA datasets. The survival analysis highlights the prognostic significance of CLDN22. [file IJOG-2025-9367254-s004.png]
